# Green synthesized plant-based silver nanoparticles: therapeutic prospective for anticancer and antiviral activity

**DOI:** 10.1186/s40486-021-00131-6

**Published:** 2021-05-03

**Authors:** Nancy Jain, Priyanshu Jain, Devyani Rajput, Umesh Kumar Patil

**Affiliations:** Department of Pharmaceutical Sciences, Dr. Harisingh Gour Vishwavidyalaya (A Central University), Sagar, M.P. 470003 India

**Keywords:** Nanotechnology, Silver nanoparticles, Green synthesis, Anticancer, Antiviral, Medicinal plants

## Abstract

Nanotechnology holds an emerging domain of medical science as it can be utilized virtually in all areas. Phyto-constituents are valuable and encouraging candidates for synthesizing green silver nanoparticles (AgNPs) which possess great potentials toward chronic diseases. This review gives an overview of the Green approach of AgNPs synthesis and its characterization. The present review further explores the potentials of Phyto-based AgNPs toward anticancer and antiviral activity including its probable mechanism of action. Green synthesized AgNPs prepared by numerous medicinal plants extract are critically reviewed for cancer and viral infection. Thus, this article mainly highlights green synthesized Phyto-based AgNPs with their potential applications for cancer and viral infection including mechanism of action and therapeutic future prospective in a single window. 
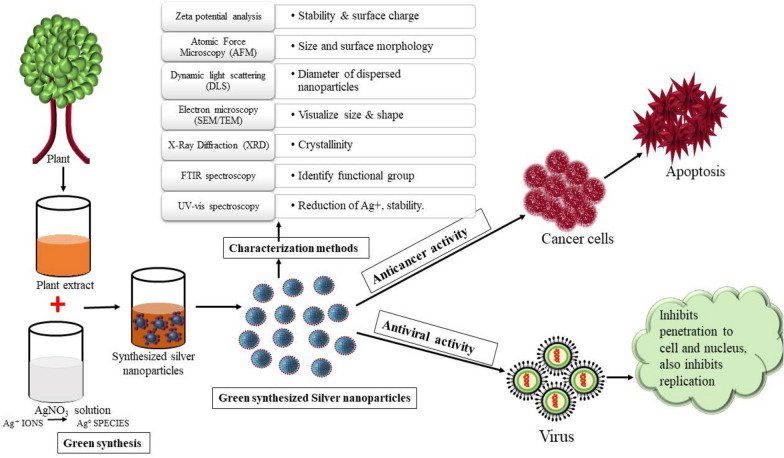

## Highlights

- Green approach of silver nanoparticle synthesis using Phyto-constituents is reviewed.
- Characterization methods of silver nanoparticles are discussed.
- Potential for anticancer and antiviral activities of Phyto-based silver nanoparticles including mode of action are highlighted.
- Therapeutic prospective and future challenges are summarized.

## Introduction

Advancement in the field of medical science is uplifted by the development of nanotechnology which provides tremendous solutions to deal with life-threatening diseases. The nanotechnology is a huge milestone which have various applications in many sectors like electronics [1], textiles [2] and most importantly in healthcare as targeted drug delivery, diagnosis, treatment, biosensing for the welfare of mankind [3]. Nanoparticles present a highly attractive platform for a diverse array of biological applications. Nanoparticles are more targeted treatments for difficult to manage diseases such as cancers.

The biggest challenge in the treatment of cancer is to prevent non-cancerous cells from destruction while damaging the tumor cells. Current mode of treatment, either oral or parenteral, circulate in the entire body and cause harm [4]. Targeted drug therapies using nanosized formulations can be a useful approach to rectify this problem and only the proliferating cancerous cells will be targeted for cytotoxicity. Nanosized formulations are truly remarkable gift for the treatment of chronic disease such as cancer [5].

The viral disease which is the cause of today’s pandemic has grown the terror to mankind and ruining the world. Millions of people lost their lives globally, while others lost their families, people lost employment, children lost their proper way of education, and this all leads to economic crises worldwide. Corona virus the ultimate villain of this epidemic [6]. Not only coronavirus but other viruses also develop and spread widely and cause life-threatening diseases like- HIV, Herpesvirus, Influenza virus, Hantavirus, Ebolavirus, Nipah virus [7]. All pharmaceutical companies and researchers are engaged to develop vaccines against the virus. Nevertheless, the world can’t get over it. This alarms urgent research and development of the new antiviral drug to cure the human health of life-threatening viruses.

Metallic nanoparticles are attacking much attention because of their unique properties and use. Nanoparticles of silver metal are the most extensively studied as it offers tremendous broad-spectrum activities. Research on AgNPs has made giant strides in nanoscience especially as antimicrobial, antibacterial [8, 9], antioxidants [10], antifungal [11], anti-inflammatory [12], anticancer [13], anti-angiogenic [14], AgNPs are small in the size range of 10–100 nm with unique Physico-chemical properties (size, shape, optical activity, electric conductivity, high surface area). Plant mediated AgNPs are safe, eco-friendly, cost-effective, rapidly synthesized at the same time they play a vital role as reducing, stabilizing, and capping agents. Thus, the green method of synthesizing AgNPs offers numerous advantages over chemical and physical methods.

Silver nanoparticles are one of the most vital and fascinating nanomaterials among several metallic nanoparticles that are involved in biomedical applications [4, 5]. Silver nanoparticles have attracted increasing attention for the wide range of application in biomedicine. They are used as antimicrobial agents in wound dressings, as topical creams to prevent wound infection and as anticancer agents [8]. Nano sized metallic particles are unique and can considerably change physical, chemical and biological properties due to their surface to volume ratio, therefore these nanoparticles have been exploited for various purposes [3]. Green synthesized nanoparticles show high yield, solubility and high stability. Among several synthetic methods for AgNPs biological methods seems to be simple, rapid, non-toxic, dependable and green approaches than can produce well-defined size and morphology under optimized conditions for traditional research [5, 7].

This article is an attempt to expose greenly synthesized AgNPs overviewing their methods of characterization and application in the field of bioscience. Considering the literature in this regard, anticancer and antiviral activities of AgNPs are described with their possible mechanism of action on different cell lines. Before concluding the article, important therapeutic and future challenges of AgNPs regarding anticancer and antiviral activity were discussed.

## Green synthesis

Green synthesis is the biological method of synthesizing nanoparticles. Green synthesis of AgNPs is the most accepted method as it provides various advantages over conventional techniques (chemical and physical methods). The technique is eco-friendly, easy, no sophisticated instruments and chemicals are required. No toxic chemicals are involved as reducing agents and stabilizing agents are derived from plants [15]. Plants provide free reducing, stabilizing, and capping agent and also cost of microorganism and culture media is reduced. Ultimately reducing the overall cost of the formulation [16, 17]. This method is a good alternative to conventional methods of nanoparticles synthesis. The product formed using this method is more stable with the desired shape and size [18, 19].

**Fig.1:**
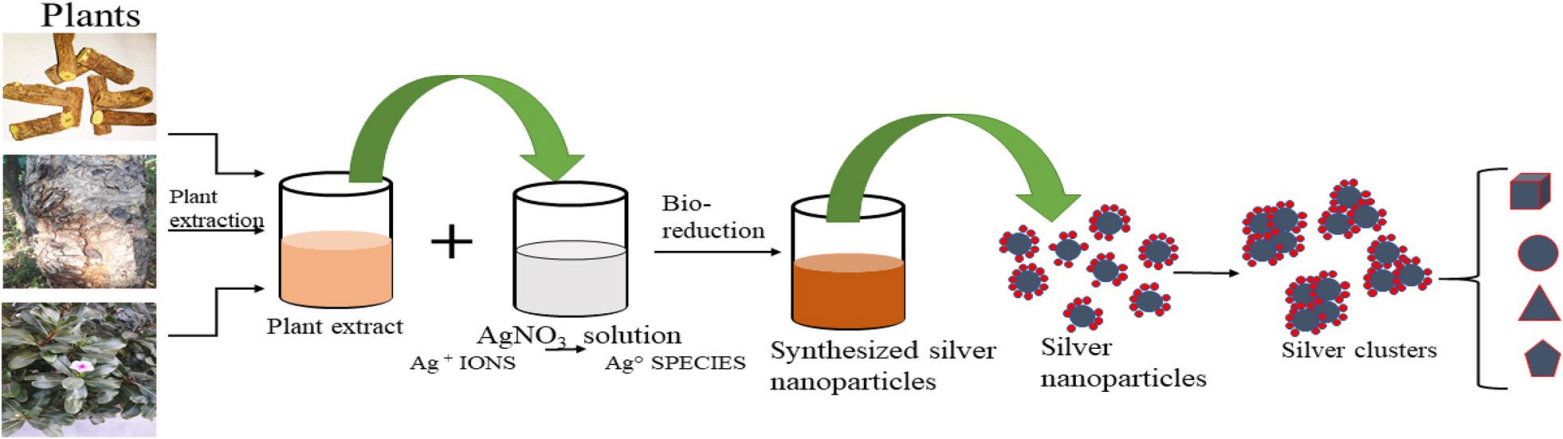
Synthesis of AgNPs through Green synthesis method

Naturally occurring phytoconstituents consist of numerous primary and secondary metabolites such as proteins, amino acid, vitamins, nucleic acids and alkaloids, terpenoids, flavonoids, saponins, phenols respectively [20]. These primary and secondary metabolites in plant extract act as reducing agents for silver ions by getting oxidized and coats the newly developed particles. In the presence of oxygen, such as in silver nitrate (AgNO_3_), these metabolites lose their electron and become oxidized via common cellular procedures, thus act as reducing agents [21, 22] (Fig. 1).

The process of green synthesis begins when the plant extract is mixed with silver nitrate solution. Over a certain period of time, the change in the color indicates the formation of nanoparticles. Silver nitrate solution which has positive ions (Ag +) converts to zero-valent state (Ag° species) when plant extract or active constituents from plants are added to it, which acts as a reducing agent. Then the nucleation process begins which is followed by the immediate growth phase. This leads to join smaller particles to form larger nanoparticles which are more stable thermodynamically. Finally, different shapes of nanoparticles are formed like cubes, spheres, triangles, hexagons, pentagons, rods, and wires. Several factors that affect the synthesis and formation of nanoparticles are pH, temperature, the concentration of plant extract, reaction time, the concentration of silver nitrate, pressure, and others [23, 24].

Phytoconstituent of the plant act as an excellent reducing and stabilizing agent. The flower extract of *Lonicera* *hypoglauca* flower act as reducing and capping agents in the synthesis of AgNPs and possesses anticancer activity [25]. *Artocarpus integer* leaf extract was used to synthesize AgNPs and formed the spherical NPs of 5.76 nm to 19.1 nm [26]. *Catharanthus* *roseus* extract used to synthesize AgNPs showed the presence of alkaloid of indole type which acts as a reducing and stabilizing agent [10]. Greenly synthesized AgNPs using leaf extract of *Clitoria ternatea* and *Solanum* *nigrum* showed antibacterial activity against nosocomial pathogens. The synthesis of nanoparticles was confirmed by UV, FTIR, SEM, and XRD [27]. *Abelmoschus esculentus* (L.) pulp extract was incorporated to form AgNPs of 3-11nm and showed anticancer and antimicrobial activity [28]. Besides these, several other types of research show well-developed nanoparticles using the green synthesis method and their potential role in medicine.

## Characterization of plant-based silver nanoparticles-

**Fig.2:**
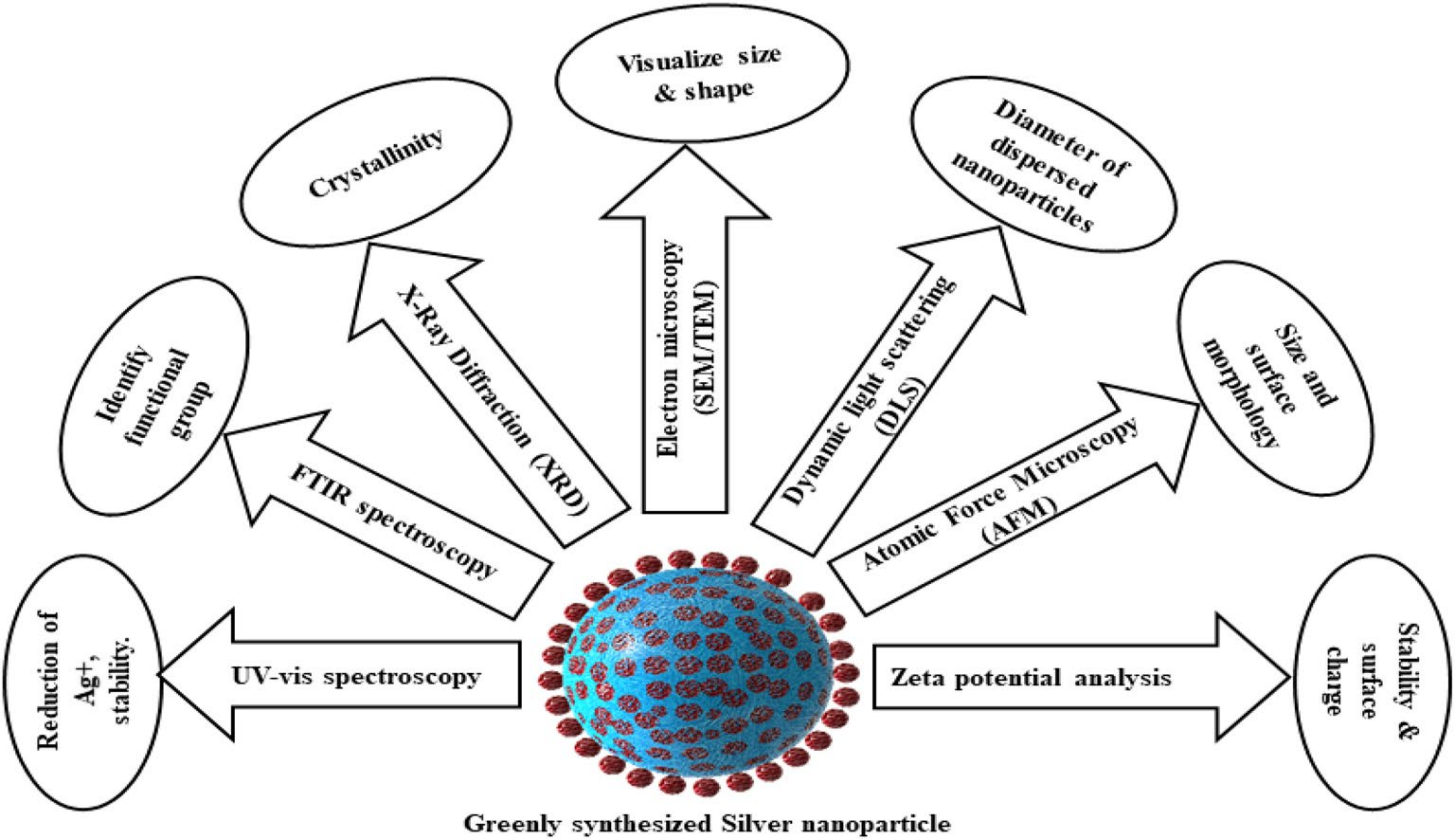
AgNPs with their Characterization Methods

Different factors modulate the characteristics of AgNPs like shape, size, crystallinity, surface charge, surface coating, and biological activity. There are several technologies available to study the characters and properties of nanoparticles such as Ultra-violet visible spectroscopy (UV-vis), X-ray diffraction (XRD), Fourier Transform Infrared (FTIR) spectroscopy, scanning electron microscopy (SEM), Transmission electron microscope (TEM), Dynamic light scattering (DLS), Atomic Force Microscopy (AFM) (Fig. 2).

### UV–vis spectroscopy

UV-Vis spectroscopy is the simple, effective, and primary characterization technique used to determine the stability, optical properties, and the synthesis reaction conditions such as time, temperature, and pH [29]. The free-electron oscillates and produces charges over the surface of nanoparticles under electromagnetic radiations as a result of the SPR effect [20]. The process of AgNPs synthesis is the coloured reaction and shows strong and sharp absorption bands under the visible region in the range of 400–500 nm [30]. Curcumin loaded AgNPs synthesized with different concentration of pure curcumin as 0.005 g (C0), 0.1g (C1), and 0.25g (C2) showed absorbance spectra at 427 nm, 428 nm, and 445 nm for C0, C1, C2 respectively [31]. *Salvia spinosa* grown extract loaded green synthesized nanoparticles has shown broad bell-shaped spectrum curve in UV-Vis analysis [32]. Similarly, the change in the color of the reaction and reduced silver ions can and has been measured using UV-Vis spectroscopy in many studies [33–36].

### X-Ray diffraction (XRD)

XRD is a characterization methodology for measuring the crystallinity of the AgNPs. X-rays strike the crystal surface and interact with the atoms. The atoms arrange themselves at a proper distance on the crystalline plane and show a pattern of diffraction [20, 30]. XRD characterization method is been used in different researches to determine the crystallinity of green synthesized AgNPs. AgNPs prepared using aqueous leaf extract of *Urtica dioica* Lin resulted crystalline structure by showing average particle size ~25 nm. The sample show strong reflection at 38.45°, 46.35°, 64.75°, and 78.05° that attributes to 111, 200, 220, and 311crystalline plane [37]. Similarly, XRD pattern of AgNPs prepared using *Pedalium murex* leaf extract showed peaks at 38.19°, 44.37°, 64.56° and 77.47° attributes to the crystalline plane of 111, 200, 220, and 311 with average size of 14nm [38]. The XRD pattern obtained from the silver nanoparticles synthesized using the leaf extract of *Clitoria ternatea* has shown intense peak at 28, 33, 38, 44, 46, 55, 58, 65 and 77 and silver nanoparticles synthesized using the leaf extract of *Solanum nigrum* has peak at 28, 32, 39, 45, 55, 57, 65, 69, 75 and 77 which are induced as crystalline silver [27].

### Fourier transform infrared (FTIR) spectroscopy

FTIR is a highly reliable analytical method that detects and displays elements, chemical structure, chemical bonds, functional groups, and bonding arrangements of molecules [9, 39]. AgNPs characterization through FTIR is done to identify the molecules which act as coating and stabilizing agents and also to detect the reduction of silver ions [20]. The FTIR spectra shows that amide and carboxylic functional groups may be responsible for the reduction or capping in the green synthesis of AgNPs [30]. Greenly synthesized AgNPs using leaf extract of *Catharanthus roseus* shows major peaks at 2401, 2073, 1706, 1084, and 8208 cm^-1^ which indicates the presence of different functional groups such as carboxylic acid group (O-H), Alkynes group (RC=CH), ketone group (C=O), Alcohol and amide groups, and phenyl ring, primary and secondary amine (N-H) group respectively [9]. *Tectona grandis* seeds extract loaded greenly synthesized nanoparticles FTIR spectrum showed bands at 1745, 1643, 1508 and 1038 cm-1 were assigned to stretching vibration of C=O bond of carboxylic acid or ester, N-C=O amide bond of proteins, nitro compounds, C-N amine bond respectively [40].

### Electron microscopy

Electron microscopy is the high-resolution microscopy and the most accepted method to determine the morphology of the nanoparticles. This includes scanning electron microscopy (SEM) and Transmission Electron microscopy (TEM). The greenly synthesized AgNPs can be visualized when the electron beam strikes the nanostructured particles. Structural characterization of AgNPs using electron microscopy provides qualitative and quantitative information regarding the size, shape, size distribution, dry diameter distribution [20, 30].

#### Scanning electron microscopy (SEM)

SEM visualize the surface morphology of the sample. The image is obtained when the electron is reflected from the surface of the sample [20]. The high-resolution image of the surface of nanoparticles which enrich us with valuable information like size, shape, topography, composition, electrical conductivity, and other properties [30]. There are many examples of greenly synthesized AgNPs characterized by SEM. SEM analysis of Acetyl-11-keto-β-boswellic acid mediated AgNPs (AKBA-AgNPs) showed spherical shape AgNPs with size range of 6–70 nm [41]. Similarly, AgNPs prepared using root extract of *Glycyrrhiza glabra* and leaf extract of *Artemisia turcomanica* showed particles diameter as 20–30 nm and 21.22 nm respectively [17, 42]. FESEM of *Tectona grandis* seeds extract loaded silver nanoparticles shows the presence of oval, spherical shape nanoparticles. The AgNPs were in the range of 10–30 nm and confirms the face centred cubic (fcc) crystalline structure of metallic silver [40].

#### Transmission electron microscopy (TEM)

TEM provides the direct visualizes of the image which is obtained from the transmitted electron. It gives the structural and chemical behavior of the nanoparticles at a high electron beam with high resolution [20]. Greenly synthesized AgNPs have been characterized and visualized using TEM by many researchers. AgNPs prepared using leaf extracts of *Viburnum lantana, Couroupita guianensis,* and *Malachra capitata* resulted in size range of 20–70 nm, 25–40 nm, 30–35 nm respectively and possess predominantly spherical shape [43–45]. *Lysiloma acapulunsis* extract loaded silver nanoparticles TEM analysis showed the crystalline structure with visible lattice fringes [46]. The photographic image is formed when the sample and the high-intensity electron beam interact with each other. It is the most accepted technique to study the formation of AgNPs by directly visualizing the image of the nanoparticles. It has a unique ability to detect the core structure, diameter, size, shape, etc. [30].

### Atomic force microscopy (AFM)

AFM is also used for the analysis of the size, surface morphology, mechano-structural and physical properties by phosphorus-doped silicon probe [20]. For characterization, the sample of AgNPs is prepared by dissolving in water or ethanol and the droplet is applied to the silicon substrate and allowed to dry. After drying, AFM analysis of the silicon-substrate which consists of the sample on it is done using a probe [30]. Tamoxifen-loaded AgNPs on AFM studies showed average size range 17.5 ± 2.5 nm [47].

### Dynamic light scattering (DLS)

DLS provides the diameter of particles present in the formulation which are dispersed in the liquid. It determines the size of the AgNPs colloidal suspension. DLS is based on the principle of scattering of light. DLS is been used widely for the characterization of AgNPs which are synthesized using phytoconstituents [38, 48]. The dispersed particles in the colloidal suspension scatter the light and as a result the image of the particles is obtained and size distribution can be determined in the range of 0.3–10 µm [20]. *Pedalium murex* leaf extract mediated AgNPs showed the average particle size distribution of 73.14  nm [38]. Similarly, AgNPs synthesized using *Salvia miltiorrhiza* extract showed the particle size 128 nm [49].

### Zeta potential analysis

Zeta potential analysis is usually done to determine the surface charge and stability of the formulation. By this analysis, one can evaluate the colloidal stability of the greenly synthesized AgNPs by quantifying the velocity of the nano-sized particles. Under the influence of the electric field, the velocity of the particles is evaluated at which they travel towards the electrodes [20]. AgNPs synthesized using seed extract of *Nigella sativa* and leaf extracts of *Gloriosa superba* and *Cynara scolymus* showed that particles possess negative charge with the potential of − 18.8 ± 0.372, − 27.0, and − 32.3 ± 0.8 mV respectively [50–52]. Zeta potential of the *Phyla dulcis* extract loaded silver nanoparticles was analyzed and values were between − 20 and − 24 mV indicated that the AgNPs are relatively stable [53].

## Plant- based silver nanoparticles for cancer

Cancerous cells evade apoptosis or programmed cell death and continue to proliferate. The aforementioned is the hallmark of cancer cells and the major focus of cancer therapy development. Plant-based nanosized silver is emerging to tackle cancer effectively. Two signally pathways i.e. intrinsic pathway and extrinsic pathway that exist for the activation of programmed cell death or Apoptosis. DNA damage or severe cell stress triggers apoptosis which is depriving in cancerous cells. Greenly synthesized AgNP using a bioactive fraction of *Pinus roxburghii* were reported to possess cytotoxic activity against lungs and prostate cancer cells. Apoptosis was examined to be induced through the intrinsic pathway via mitochondrial depolarization and DNA damage. An increase in ROS, cell cycle arrest, and caspase-3 activation also leads to apoptosis of cancer cells [54]. AgNPs synthesized utilizing *Phyllanthus emblica* leaf extract showed anticancer activity against Hepatocellular carcinoma (HCC) [55]. AgNP-dipalmitoyl-phosphatidylcholine composites forming liposomes (Lipo-AgNP) were found cytotoxic by inducing ROS formation and DNA damage. Activation of proapoptotic protein Bax and inhibition of Bcl-2 protein leads to the release of cytochrome C and gradually activates caspase causing apoptosis in macrophages [56].

Biosynthesized AgNPs using phycocyanin reported antimicrobial and anticancer activity. Cytotoxic action was investigated against breast cancer cell line and Ehrlich ascites carcinoma bearing mice (IC50 − 27.79 ± 2.3 µg/mL) [57]. AgNPs of two size- 2 nm and 15 nm, were investigated for anticancer activity against MCF-7 and T-47D cells and determined to induce Endoplasmic reticulum stress via unfolded protein response (UPR) and also enhances activation of caspase 9 and caspase 7 causing cell death [58]. AgNPs are also confirmed to exhibit strong cytotoxic by arrest cell cycle at the G2/M phase. In an investigation on A549 lung epithelial cells, it is reported that AgNPs strongly downregulates protein kinase-C (PKCζ) which leads to the capitulation of the cell cycle at the G2/M phase. AgNPs are further involved in the upregulation of P-53 protein, Bax and Bid, caspase-3, generation of ROS, and downregulating antiapoptotic protein-Bcl-2 and Bcl-w [59]. *Cynara scolymus* also recognized as Artichoke, were employed to synthesize AgNPs and further research for anti-tumor activity with photodynamic therapy revealed that AgNPs modulates mitochondrial apoptosis via generation of ROS, regulates the apoptotic proteins and cause MCF7 breast cancer cells death [52]. Similarly, *Moringa oleifera* [60], *Tropaeolum majus* [61], *Punica granatum* [62], *Gloriosa superba* [51], *Teucrium polium* [63] plant extract used to synthesize AgNPs and reported to be cytotoxic against cancer cell lines. There are numerous related investigations and research that evidence that AgNPs are the potent and effective candidate for cancer therapy. The target cancer treatment is also possible using AgNPs.

### Mechanism of action

The process of apoptosis starts with several stages of apoptotic protein activation, DNA damage, mitochondrial degradation, the formation of Apoptosome, and ultimately cell shrinkage. These become the major important targets to be utilized in cancer therapy. Silver nanoparticle acts on certain target areas and shows anticancer activity.

Recent researches state that AgNPs majorly works by enhancing Reactive oxygen species (ROS), increasing oxidative stress, and DNA destruction. ROS maintains the normal cellular homeostasis which is crucial for cell survival. ROS involves in the cellular transduction signaling pathways and forms as a free-radical by-product from cellular metabolism [64]. An extreme amount of intracellular ROS cause DNA, lipid, protein damage as a mechanism for AgNPs induced toxicity [28, 65, 66]. One of the studies reveals that through reverse transcription-polymerase chain reaction (RTq-PCR) techniques pro-apoptotic gene upregulation in AgNPs treated HCT-116 cells [67]. In the process of apoptosis caspase enzymes play an important role. Up-regulation in the expression of caspase 3, caspase 8, and caspase 9 excessively increase the induction of apoptosis. AgNPs treatment to HCT-116 cells exposed up-regulation of pro-apoptotic enzymes- caspase3, 8 and 9, and also PUMA (mediator of apoptosis linked to p53) resulting to induce apoptosis [67, 68]. P53 is the protein mediator that controls and regulates stress signals related to apoptosis and cell cycle arrest [68]. AgNPs treatment on A549 lung epithelial cells indicates the up-regulation of p53 which leads to the arrest of the cell cycle at the G0-G1 phase and stops the cell division [59, 69]

Green synthesized AgNPs using *Coptis chinensis* describes the mechanism of action as AgNPs increase the expression of pro-apoptotic proteins- Bax and Bak and decrease anti-apoptotic Bcl-2 and Bcl-XL protein [70]. B-cell lymphoma-2 (Bcl2) protein is a family of pro-apoptotic protein and anti-apoptotic protein, which are involved in the regulation of apoptosis. The pro-apoptotic proteins are- Bax and Bak, which initiate and stimulate the process of apoptosis. In the class of anti-apoptotic protein include- Bcl-2 and Bcl-XL protein, which are involved in the suppression of apoptosis [71, 72].

**Fig. 3:**
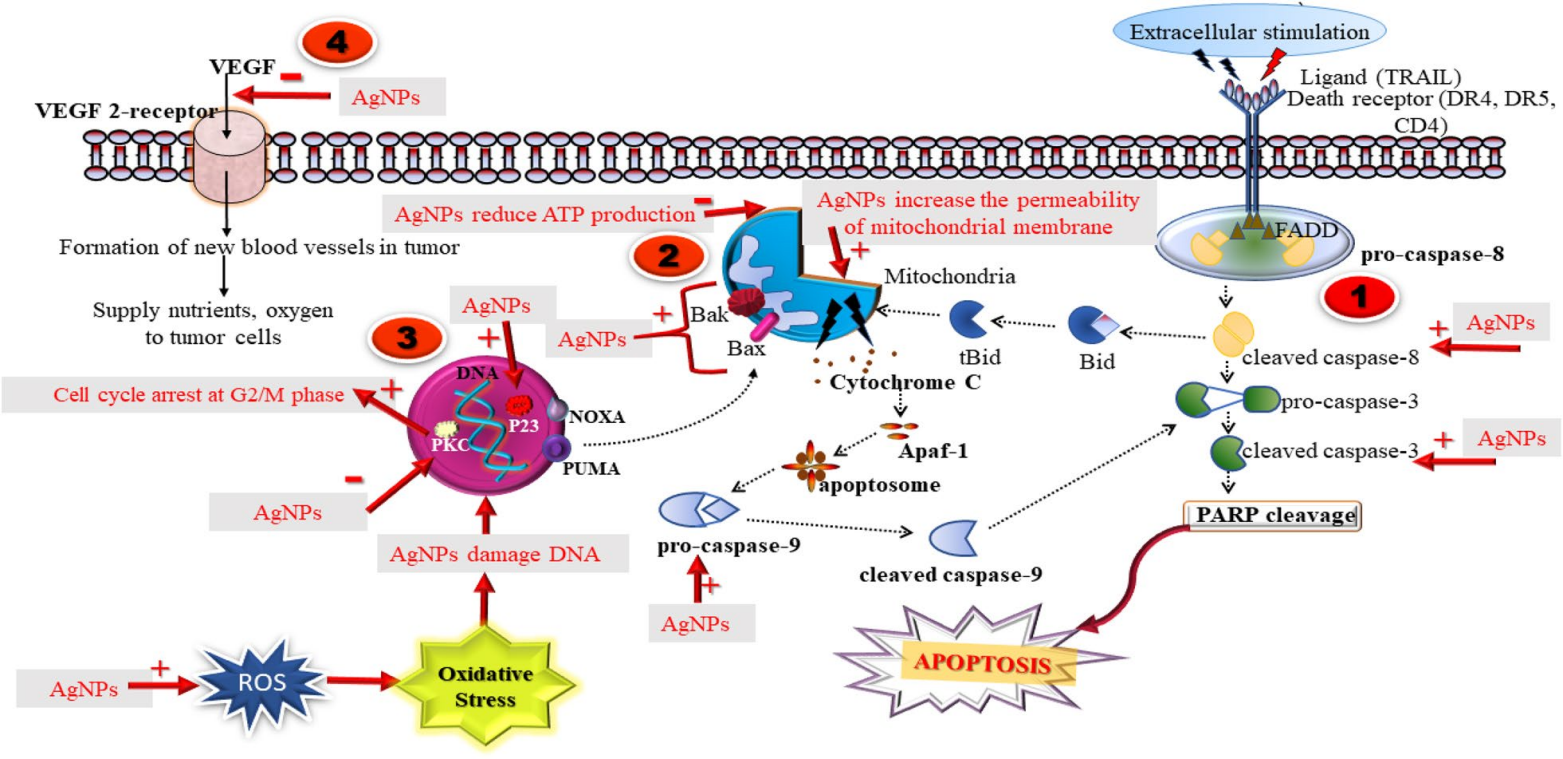
(1) AgNPs upregulates caspase-8 which leads to stimulate and activate pro-apoptotic proteins- Bid, tBid, and further increase the release of cytochrome C from mitochondria which activates Apaf-1, then forms Apoptosome. Following the formation of Apoptosome, caspases are activated and result in apoptosis. AgNPs also directly upregulates cleaved caspase-3 to increase the process of apoptosis. (2) AgNPs results in mitochondrial apoptosis by increasing the permeability of the mitochondrial membrane and reducing the production of ATP. AgNPs also upregulates the apoptotic protein- Bak and Bax, which increase the release of cytochrome C and induce apoptosis. (3) AgNPs acts on genetic material DNA and causes damages by the increasing generation of ROS that causes oxidative stress and damages the DNA. These nanoparticles inhibit the protein kinases (PKC) and cause cell cycle arrest at the G2/M phase. AgNPs upregulates P-23 Protein which is responsible to activate the apoptosis process by activating other pro-apoptotic protein. Following the DNA damage intracellular pro-apoptotic proteins are released to activate caspases and cause cell death. (4) Vascular endothelial growth factor (VEGF), which is the pro-angiogenic factor involved in the activation of signaling pathways that promotes cell proliferation and migration through tyrosine kinase receptor (VEGFR2) and cause angiogenesis in tumor cells. This mechanism is inhibited by AgNPs by suppressing VEGF induced cell proliferation

Vascular endothelial growth factor (VEGF) causes angiogenesis which can proliferate the tumor cells and can transform the tumor from benign to a malignant state. Angiogenesis is the formation of the new blood vessel from the existing blood vessels. This is promoted by VEGF, which acts as a pro-angiogenic factor via VEGF-2 receptor (tyrosine kinases) [73, 74]. The mechanism of action of AgNPs is extensively elaborated in an investigation that reveals VEGF-induced proliferation by angiogenesis is suppressed by AgNPs. AgNPs are considered as the potent anti-angiogenic agent that inhibits VEFG [14, 74]. AgNPs activate apoptosis through cellular damages, anti-angiogenic pathway, and caspase cascade pathway. Schematic mechanism of action of AgNPs for anticancer activity is depicted in Fig. 3. Various Plant-based Silver Nanoparticles have been developed for various anticancer activity. Their mechanism of action and other findings including cell model used for evaluation are summarised in Table 1.

**Table 1:** Plant-based Silver Nanoparticles for anticancer activity

Type of cancer	Plant	Part	Extract used	Characterization	Shape	Size	Cell model	MOA	Doses	References
Human breast cancer	*Artocarpus integer*	Leaf	Aqueous extract	UV–Visible, FTIR, TEM, TGA	Spherical	5.76 nm to 19 nm	MCF-7, MG-63 cells	Interfere with DNA, cell damage, cytotoxicity	70–90 µg/ml	[26]
	*Annona squamosa*	Leaf	Aqueous extract	UV–vis., FTIR, XRD, TEM, EDS, zeta potential	Spherical	20 to 100 nm	MCF-7 cells	Induction of apoptosis	IC_50_-30–50 g/ml	[75]
	*Cynara scolymus*	Leaf	Aqueous extract	FTIR, SEM, XRD, UV–Vis	Spherical	98.47 ± 2.04 nm	MCF7 cells	Mitochondrial apoptosis	IC_50_-10 mg/mL	[52]
	*Camellia Sinensis Green tea*	Leaf	Aqueous solution	FTIR, SEM, XRD, UV–Vis, AFM	Spherical	~ 420 nm	MCF-7 cells	Decrease O-GlcNAc Transferase (OGT) and cytotoxicity	5–40 µg/mL	[47]
	*Couroupita guianensis (Cannonball)*	Leaf	Aqueous extract	UV–Vis, FTIR, SEM, TEM	Spherical, triangle	25 to 40 nm	MCF-7 Cell Line	Cytotoxicity	IC_50_-20μL/mL	[44]
	*Glycyrrhiza uralensis*	Roots	Aqueous extract	UV–Vis, FTIR, XRD, SEM, TEM, DLS	Spherical	5-15 nm	MCF7cells	Cytotoxicity	10lg/mL	[76]
	*Juglans regia (Walnuts)*	Walnut Fruits- Husks	Aqueous extract	FTIR, SEM, UV–Vis XRD	Spherical	31.4 nm	MCF-7 cell	Disturb the signaling pathway, increase ROS	60 µg/mL	[77]
	*Lonicera hypoglauca*	Flower	Aqueous extract	UV–Vis, FTIR, SEM–EDS, TEM & SAED	Spherical, some rod-hexagonal	4.99 to 25.83 nm	MCF-7 cells	Increases in expressions of pro-apoptotic Bax, caspase-3 & caspase-9	–	[25]
	*Sesbania grandiflora*	Leaf	Aqueous extract	UV–Vis, FTIR, field emission SEM,	Spherical	22 nm	MCF-7	Apoptosis, oxidative stress, interfere cell membrane integrity, decrease cell proliferation, DNA damage	IC_50_-20 µg/ml	[78]
	*Melia dubia*	Leaf	Aqueous extract	UV–visible, XRD and SEM–EDS	Spherical & irregular shape	7.3 nm	KB cell line	Cell toxicity	IC_50_-31.2 lg/ml	[79]
	*Nostoc linckia*	Cyanobacterium	Phycocyanin-Protein extract	TEM, FTIR, UV–Vis,	Spherical	9.39 to 25.89 nm	MCF-7	Inhibits growth of tumor	IC_50_-27.79 ± 2.3 µg/mL	[57]
Human T-cell lymphoma	*Abelmoschus esculentus*	Pulp	Aqueous extract	UV–vis., TEM, XRD, FTIR	Spherical	6.7 nm	Jurkat cell line	Increase of ROS, nitrogen species, loss of integrity of mitochondria	IC_50_-6.15lg/ml	[28]
Prostate cancer cells	*Alternanthera sessili*	Leaf	Aqueous extract	FTIR, SEM, UV–Vis XRD	Spherical	30–50 nm	PC3	Antiproliferative, cell toxicity	6.85 μg/ml	[80]
	*Salvia miltiorrhiza*	Leaf	Aqueous extract	UV–Vis, FTIR, XRD, SEM, EDX	Spherical, oval, hexagonal triangular	80 and 12 nm	LNCaP cell lines	Apoptosis and cytotoxicity by bax, Bcl2 intrinsic pathway	50 mg/ml	[49]
Gastric cancer cell line	*Artemisia turcomanica*	Leaf	Ethanolic extract	UV–Vis, FTIR, XRD, SEM, TEM	Spherical	20-60 nm	AGS	Induce apoptosis and cytotoxicity	14.56 μg/ml	[17]
Lung carcinoma	*Bauhinia tomentosa Linn (Kanchini)*	Leaf	Aqueous extract	UV–Visible, FTIR, FESEM-EDAX, HR-TEM and XRD	Spherical	11.6 nm to 33 nm	A-549	Inhibits proliferation	28.125 μg/mL	[81]
	*Capparis zeylanicu*	Leaf	Aqueous extract	UV–Vis, FTIR, SEM, TEM, XRD	Spherical	28 nm	A549 cell line	Induce apoptosis	1.63–200 μg/ml	[82]
	*Coptis chinensis*	Leaf	Methanolic extract	FTIR, SEM, XRD, UV–Vis	Smooth spherical	6–45 nm	A549-cell	Induce intrinsic pathway apoptosis	10 µg/mL and 25 µg/mL	[70]
	*Indigofera tinctoria*	Leaf	Aqueous extract	UV–vis., FTIR, XRD, TEM, EDX, AFM	Spherical	9 nm to 26 nm	A549	Increase ROS and leads to cell death	56.62 ± 0.86lg/ml	[83]
	*Zanthoxylum rhetsa*	Seed Coat	Aqueous extract	UV–Vis., AFM, TEM, SEM, EDX, XRD and FTIR	Spherical	10 nm to 68 nm	A549	Ag^+^ ion interact with cell membrane, protein, DNA, RNA leads to cell death	IC_50_—65.17 μg/ml	[84]
Hepatic cancer	*Asafoetida*	Gum	Aqueous extract	UV–vis., TEM, SEM, DLS	Spherical	90–95 nm	HepG2 cell line	Antiproliferative	–	[85]
	*Myrtus communis*	Aerial Parts	Aqueous extract	UV–Vis, FTIR, EDX, TEM & XRD	Spherical	20–30 nm	HepG2	down regulation of Pl3k/Akt, ERK and NF-kB pathways and inhibit cell proliferation	IC_50_ -7.75 µM/mL	[86]
	*Nigella sativa*	Seeds	Aqueous extract	UV–Vis, FTIR, XRD, SEM DLS, zeta potential	Spherical	10–20 nm	HepG2 cell lines	Apoptosis & increase ROS production	IC_50_-7.16 µg/ml	[50]
	*Phyllanthus emblica*	Leaf	Aqueous extract	UV–vis., FTIR, XRD, TEM, EDX, AFM	Agglomerated spheres	38–50 nm	HeLa cells, HUH-7	Cytotoxicity	IC_50_-31.99 µg/mL	[55]
	*Taraxacum officinale (dandelion)*	Leaf	Aqueous extract	HR-TEM, UV–Vis, FTIR, XRD	Spherical	5 and 30 nm,	HepG2		IC_50_-60 μg/mL	[87]
Human skin cancer	*Boswellia serrata*	Bark	Methanolic extract	UV–Vis, FTIR, XRD, SEM, DLS	Hexagonal cubic	20.5 ± 0.5 nm	G361	–	10-4-10–7 M	[18]
	*Gelsemium sempervirens,*	Whole plant	Ethanolic extract	TEM, FTIR, UV–Vis, XRD, DLS, AFM	Spherical	90.87 nm	A375	G2/M phase arrest, effect cellular entry	IC_50_-80 µg/mL	[88]
	*Hydrastis canadensis*	Whole plant	Ethanolic extract	TEM, FTIR, UV–Vis, XRD, DLS, AFM	Spherical	90.87 nm	A375	G2/M phase arrest	IC_50_-100 µg/mL	[88]
	*Phytolacca decandra*	Whole plant	Ethanolic extract	TEM, FTIR, UV–Vis, XRD, DLS, AFM	Spherical	90.87 nm	A375	G2/M phase arrest	IC_50_- 78 µg/mL	[88]
	*Thuja occidentalis*	Whole plant	Ethanolic extract	TEM, FTIR, UV–Vis, XRD, DLS, AFM	Spherical	90.87 nm	A375	G2/M phase arrest	IC_50_-120 µg/mL	[88]
Cervical cancer	*Iresine herbstii*	Leaf	Aqueous extract	FTIR, SEM, XRD, EDX	Cubic shape	44-64 nm	HeLa	Cytotoxic activity	LC_50_-51 μg/mL	[89]
	*Moringa oleifera*	Stem Bark	Aqueous extract	HRTEM, UV–vis., DLS, FTIR, AFM, SEM	Pentagon	40 nm	HeLa	reactive oxygen species (ROS)	250 g/mL c	[60]
	*Nepeta deflersiana*	Aerial Part	Aqueous extract	UV–Vis, FTIR, XRD, SEM, EDX	Face-centered-cubic structure	33 nm	HeLA	Increase oxidative stress, apoptosis and necrosis	10–50 µg/ml	[90]
	*Nothapodytes nimmonian*	Fruit	Aqueous extract	UV–Vis, FTIR, SEM, EDX, XRD, zeta potential	Spherical	44-64 nm	HeLA cell	Inhibits proliferations	IC_50_- 87.32 ± 1.42 μg/mL	[91]
	*Punica granatum Pomegranate*	Leaf	Aqueous extract	UV–vis, FTIR, SEM, EDS, XRD, Zeta potential, FTIR	Spherical	41.69 nm to 69.61 nm	HeLa cell line	Apoptosis	IC_50_- 100µgml − ^1^	[62]
	*Prunus domestica*	Fruit- Gum	Gum solution	UV–Vis, FTIR, SEM, EDX, XRD	Spherical	5–30 nm	HeLa	Cytotoxic	IC_50_- 3.45 ± 0.23 μg/mL	[92]
Colon Cancer	*Commelina nudiflora*	Leaf	Aqueous extract	Particle Size Analyzer and Zeta Potential Study, TEM	Spherical, triangular, rod	24–150 nm	HCT-116	Upregulate apoptotic genes, apoptosis	IC_50_—100 μg/ml,	[67]
	*Flavonoids*		Aqueous solution	UV–vis, FTIR, SEM, DLS, XRD, FTIR	Spherical	2–10 nm	HCT116	Mitochondrial impairment, DNA damage	5 µg/mL	[48]
	*Vitex negundo*	Leaf	Methanol extract	UV–visible FESEM, EDX, TEM, XRD, FTIR	Spherical	5 to 47 nm	HCT15	Cell cycle arrest at G0/G1-phase, DNA damage, apoptosis	IC_50_—20 g/ml	[93]
Breast cancer, liver cancer,	*Cucumis prophetarum*	Leaf	Aqueous extract	UV–Vis, FTIR, SEM, TEM	Spherical, granulated, ellipsoidal	–	A549, MDA-MB-231, HepG2, and MCF-7	Apoptosis, cytotoxicity	105.8 μg/mL for A549, 81.1 μg/mL for MDA-MB-231, 94.2 μg/mL for HepG2, and 65.6 μg/mL for MCF-7	[94]
*Lymphoma*	*Gloriosa superba*	Leaf	Methanolic extract	UV–vis, FTIR, TEM, DLS, Zeta potential, XRD	Spherical	20–69 nm	DLA tumor cell	Cytotoxicity	ED_50_- 80 µg	[51]
Ehrlich ascites carcinoma (EAC) and human colorectal adenocarcinoma	*Clerodendrum phlomidis*	Leaf	Aqueous extract	UV–vis., FTIR, XRD, FESEM, EDX, AFM	Spherical	23–42 nm	HT29 cells & EAC cell	Free radical production, cell damage	IC_50_ – 36.72 μg/ml for HT29 cells & 32.69 μg/ml for EAC cell	[95]
Human head and neck carcinoma cells	*Glycyrrhiza glabra*	Rhizome, Root	Aqueous/ methanolic extract	UV–Vis, FTIR, SEM	Spherical	46 nm	HeLA Cells, HN-30	Activation of caspase 3	10 μg/ml,	[96, 97]
Breast and prostate cancer	*Hyptis suaveolens*	Leaf	Aqueous Callus Extract	FTIR, SEM, TEM, EDS, UV–Vis XRD	Spherical	12 to 25 nm	MDA-MB-231 and PC-3 Cells	Interfere with protein, nitrogen base, DNA and cause apoptosis	74.66 and 173.21 μg/mL	[98]
Cervical cancer cell line breast cancer cell lines	*Jurinea dolomiaea*	Leaf	Aqueous extract	FTIR, SEM, TEM, EDS, UV–Vis XRD	Spherical	28- 40 nm	HeLa & MCF-7	Apoptosis	55 ± 0.51 µg/mL	[99]
Human epithelial carcinoma	*Melia azadirachta*	Leaf	Aqueous extract	UV–visible SEM, DLS, XRD, Zeta potential	Cubical and Spherical	78 nm a	HeLa cell line	Cytotoxic effect	IC_50_-300 g/mL	[100]
Prostate & Breast adenocarcinoma	*Momordica cymbalaria*	Tuber	Aqueous extract	UV–vis., FTIR, XRD, TEM, EDX, AFM	Spherical	10–50 nm	PC-3 & MDA-MB 231 cells	Damage genetic material, cell organelle- leads to cell death	IC_50_ -72.39 and 64.03 lg/ml	[101]
	*Momordica cymbalaria*	Fruit	Aqueous extract	UV–vis., FTIR, XRD, TEM, EDX, AFM	spherical	10–50 nm	PC-3 & MDA-MB 231 cells	Damage genetic material, cell organelle- leads to cell death	IC_50_ -85.42 and 111.74 lg/ml,	[101]
Human breast cancer cell line & human colorectal adenocarcinoma cell line	*Nigella arvensis*	Seed	Aqueous extract	UV–Visible, FTIR, TEM and XRD	spherical	2–15 nm	MCF7 & HT-29	Proliferation inhibition	100 μg/mL	[102]
Lung adenocarcinomas, prostatic small cell carcinomas	*Pinus roxburghii*	Pine Needles	Methanolic extract	UV–vis., FTIR, XRD, EDX, AFM, SAED, HRTEM, FESEM	Spherical	80 nm	A549, PC-3	Induce apoptosis via caspase-3 and PARP-1 activation	IC_50_ -11.28 ± 1.28 μg/ml, 56.27 ± 1.17 μg/ml	[54]
Breast cancer, skin cancer, Leukemia	*Pueraria tuberosa*	Tubers	Aqueous extract	UV–Vis, DLS, FTIR, SEM, TEM, EDS and XRD	Spherical	162.72 ± 5.02 nm	MCF-7, MDA-MB-231, SKOV-3, U-87 and NCI/ADR cell lines	Increased cytotoxicity	3.859, 1.128, 29.36, 6.053 and 25.49 mg/ml	[103]
Human gastric cancer cell line	*Teucrium polium*	Aerial Part	Aqueous extract	UV–Vis, FTIR, SEM, XRD	Spherical	70 to 100 nm	MNK45	Provoke cell death	12.5–130 μg/mL	[63]
Skin melanoma cells & human lung cancer cells	*Carpesium cernuum*	Whole Plant	Methanolic extract	DLS, FTIR, SEM, TEM, EDS, UV–Vis, XRD	Spherical	13.0 ± 0.2 nm	B16F10 & A549	Induce apoptotic cell death	25–100 g/mL	[104]

## Plant-based silver nanoparticles for viral-infection

**Fig. 4:**
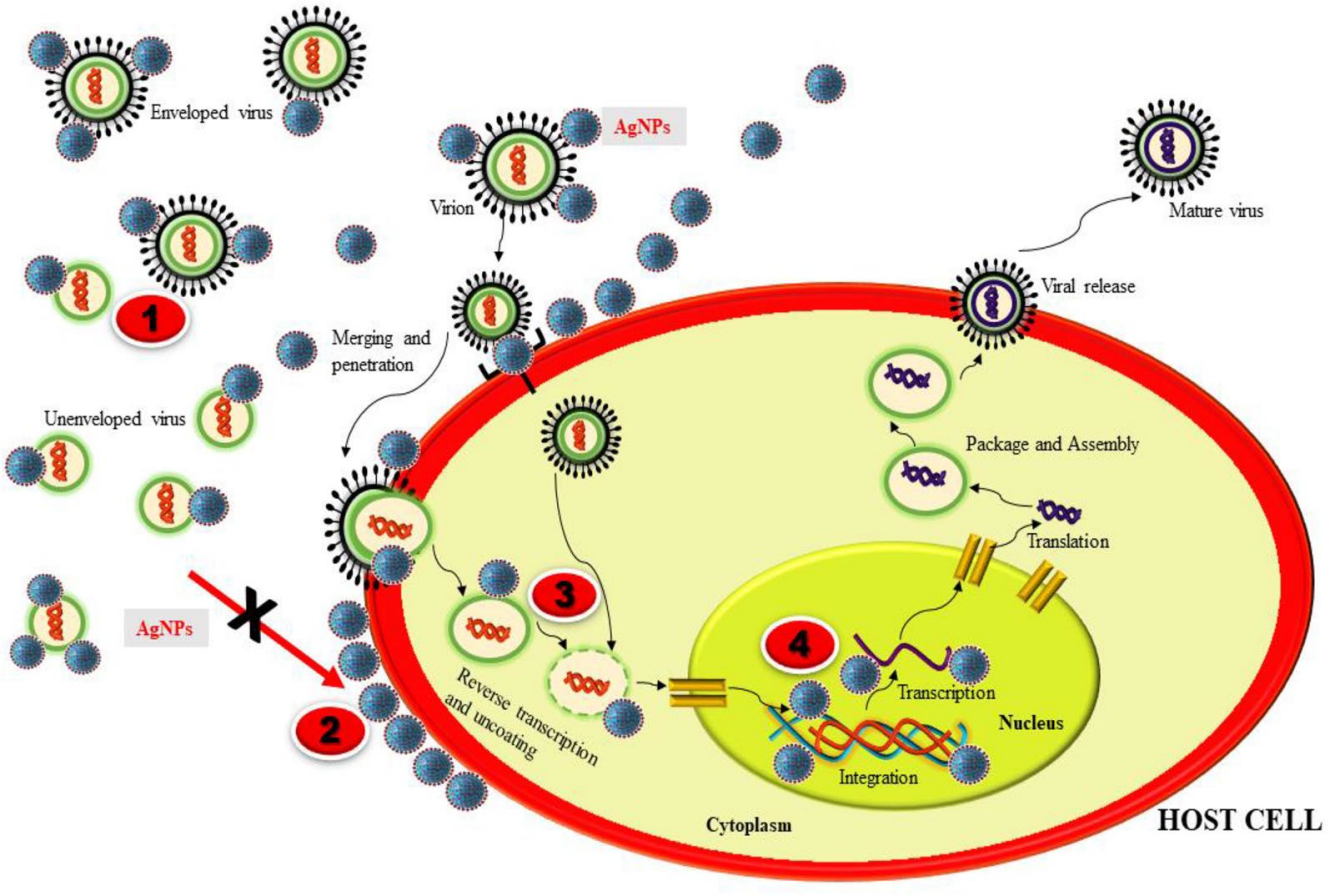
(1) AgNPs interact with the viral surface protein (gp120) in enveloped and unenveloped virus. (2) AgNPs blocks the penetration of virus into the host cell. (3) AgNPs blocks the cellular viral entry to Nucleus. (4) AgNPs inhibits the viral replication by blocking viral genome

The viral infection is a complicated infection to treatment as a virus multiply and spread quickly. Various emerging life-threatening viruses already exist overpowering humans which involve coronavirus, Ebola virus, dengue virus, HIV, Influence virus. There is an increase in studies on AgNPs as an efficacious antiviral agent. The mode of antiviral action of AgNPs, as described in various studies could be- intracellular by blocking viral replication or extracellular by interacting with viral protein (gp120) and blocking the entry which could be different for a different type of virus (Fig. 4). AgNPs are considered to the potent and novel pharmacological agent possessing effective antiviral activity against feline coronavirus (FCoV) [105], Influenza virus [106], HIV [107], Adenovirus [108], Herpes simplex virus [109], Dengue virus [110, 111], Chikungunya virus [112], Norovirus [113], bovine Herpesvirus [114], Human parainfluenza virus type 3 [115].

### Mechanism of action

Mode of action of AgNPs as viricidal in HIV-1, is described as AgNPs targets the gp120 and inhibits binding to host cell membrane. This leads to blocking entry, fusion, and infectivity [116]. The schematic mechanism of action of AgNPs for antiviral activity is depicted in Fig. 4. AgNPs interferes with the Viral replication and inhibits the release of new virus progenies at non-toxic dose 10–25 µg/ml in the size range of 10nm in a study against Tacaribe virus (new world arenavirus) [117]. The envelope of the H3N3 influenza virus consists of two main glycoprotein- Hemagglutinin and Neuraminidase. AgNPs were tested for the inhibition of Hemagglutinin glycoprotein in an investigation. The hemagglutinin is the main protein that binds to the host membrane receptor. AgNPs inhibit Hemagglutinin through interfering with the disulfide bond present on the molecule and protect the host cell by inhibiting viral genome entry and fusion [118].

In an investigation against Herpes simplex virus type-1 (HSV-1), AgNPs capped with mercaptoethane sulfonate at 400 µg/ml completely block HSV-1 infection [119]. AgNPs also inhibit early phase replication of HSV-2 at a non-toxic concentration of 100 µg/ml in VERO cells. The study also revealed that at a low dose of 6.25 µg/ml, the AgNPs could inhibit the new progeny release and at a high dose of 100 µg/ml viral replication is inhibited. It was also suggested to coat Vero cells with polysaccharides to protect the cells from AgNPs cytotoxic effects [109]. Another study on herpes simplex virus and human parainfluenza virus type 3 using biologically synthesized AgNPs clarify that AgNPs interfere and decrease replication of virus depending upon the size and zeta potential of AgNPs [115].

The size-dependent interaction of AgNPs (1–10 nm) against the HIV-1 virus was investigated in research work. The study revealed that AgNPs act as viricidal against the virus by inhibiting the binding of the virus to host cells through interacting with gp120 protein of virus envelop [107]. AgNPs synthesized using marine actinomycetes possess antiviral activity against new castle viral disease. Nanoparticles of 1–10 nm size are said to interact with gp120 and may inhibit the binding of the virus to cells [120]. AgNPs loaded with curcumin were studied antiviral activity against Respiratory syncytial virus infection (RSV). Infected Hep-2 cells were treated with Curcumin loaded AgNPs showed inactivation of the virus. The study suggested that Curcumin loaded AgNPs inhibits the entry of RSV into the Hep-2 cells i.e. blocks the attachment [121]. The study of AgNPs coated magnetic hybrid colloid, against Bacteriophage fX174, murine norovirus (MNV), adenovirus serotype-2 (AdV-2) describes that the fore-mentioned complex showed interaction with the viral surface and might damage viral protein [122]. Similarly, AgNPs-chitosan composites antiviral activity against the influenza virus [123]. Various Plant-based Silver Nanoparticles have been developed for antiviral activity. Their mechanism of action and other findings including cell model used for evaluation are summarised in Table 2.

**Table 2:** Plant-based Silver Nanoparticles for antiviral activity

Method of synthesis of AgNPs	Type of virus	Characterization	Shape	Size	Viral model	Assays/ Evaluation parameters	MOA	Doses	References
Biological synthesis using fungi	Herpes Simplex Virus & Human Parainfluenza Virus Type 3	TEM, UV–Vis, zeta potential	Spherical	46 nm and 40 nm	VERO cells	MTT assay, cotreatment assay, cell pretreatment assay, cell post-treatment assay, Virus pretreatment assay	Inhibits viral replication	ID_50_-10 mg/mL,	[115]
Biological synthesis using seaweeds	HSV-1 and HSV-2	UV–Vis, FTIR, XRD, TEM,	Spherical	8-27 nm	Vero cells	Cytotoxic assay, antiviral assay	Cytotoxic	ID_50_-2.5 μL	[124, 125]
Biological synthesis using Bacteria (*Bacillus* species)	Bean Yellow Mosaic Virus	UV–VIS, EDX, TEM, DLS, FTIR	Triangular, Hexagonal and Spherical	77–92 nm	Seeds of *Vicia faba*	RT-PCR, ELIZA	Inhibit the growth of virus	–	[126]
Biological synthesis (Bio-reduction)	Bombyx mori Nuclear Polyhedrosis virus (BmNPV)	HR-SEM, EDAX, TEM, AFM CM	Hexahedron	0.87–1.2 µm	Silkworm (*Bombyx mori)*	SDS-PAGE analysis, Energy Dispersive X-ray Analysis	Interact with cell membrane of virus	–	[127]
Chemical synthesis (citrate-stabilized AgNPs)	Feline Calicivirus	SEM, Mass spectroscopy, DLS,	Spherical	10, 75, and 110 nm	FCV strain 2280 (ATCC-VR-2057) and Crandell-Rees feline kidney (CRFK) cells	Infectivity assay, Western blot analysis, Cytotoxic assay, SDS-PAGE analysis,	Cytotoxic, viricidal	25, 50, & 100 lg/ mL	[113]
Chemical synthesis (non-surface capped AgNPs)	Vaccinia virus	TEM, XRD	Spherical	25 nm ± 10 nm,	VERO 76, BS-C-1, HeLa	Plaque Assay, VACV Adsorption Plaque Assay, Beta-Galactosidase Assay, Viral Entry Assay, VACV Adsorption Confocal Assay, MTT Assay, Dextran Uptake Assay, Western Blot	Macropinocytosis, interferes the entry	27.4 ± 3.3 µg/ml	[128]
Chemical synthesis—Polyol method (PVP-coated AgNPs), carbon-coated AgNPs, bovine-serum AgNPs	HIV-1	TEM, STEM, UV–Vis, EDS,	Spherical	PVP-coated- 6.53 nm Carbon-coated- 16.19 nm Bovine-serum AgNPs- 2.08 nm	MT-2, cMAGI HIV-1 cells	Inhibition of HIV-1 with AgNPs analysis	Interact with the glycoprotein of virus,	25 µg/mL	[107]
Chemical synthesis using Tannic acid	Adeno virus type 3	TEM	Hexahedron	70-90 nm	HeLa cells	Cytotoxicity test, MTT assay, immunofluorescence analysis, RT-PCR	Interact with DNA,	9.3 µg/mL	[108]
Chemical synthesis using citrate, PVP, H_2_O_2_	bovine herpesvirus-1	TEM, UV–Vis, zeta-sizer,	Spherical	20–25 nm	MDBK cells	Cytotoxicity assay, colorimetric-based assay, Anti-BoHV-1 effect of Ag-NPs, cytopathic effects analysis,	Attach to glycoprotein and inhibit viral normal functioning	24 µg/mL	[114, 129]
Chemical synthesis (uncoated and polysaccharide coated AgNPs)	Tacaribe virus (TCRV)	TEM	Spherical	10 and 25 nm	Vero cells	Viral Inhibition Assay, S segment real time PCR, Post-infection treatment with Ag-NPs study	Inhibits early stage of viral replication	25 μg/ml	[117]
Chemical synthesis using chitosan as stabilizer	African swine fever virus (ASFV)	TEM, UV–Vis,	Spherical	14 nm	Primary porcine alveolar macrophages (PAMs)	Cell toxicity test, Antivirus activity	Strong antiviral activity	0.78 ppm	[130]
Chemical synthesis (coated PVP)	Respiratory syncytial virus (RSV)	SEM, TEM	Spherical	8–12 nm	A549 cells (a human alveolar type II-like epithelial cell lines) & HEp-2 Cell	qRT-PCR assays, Airway Obstruction analysis, ELISA, plaque assay	Interact with glycoproteins, prevent fusion	50 µg/mL	[131]
Chemical synthesis (curcumin modified AgNPs)	Respiratory Syncytial Virus Infection	DLS, XPS, UV/vis, TEM, SEM, FTIR, zeta-sizer,	Spherical	11.95 ± 0.23 nm	Hep-2 cells	Tissue culture infectious doses (TCID50) assay, Viral titer assay, Plaque assay, Indirect immunofluorescence assay, cytopathic effect analysis, RT-PCR analysis,	Inactivate virus, inhibits entry into host cell		[121]
Chemical synthesis (PVP coated)	Herpes simplex virus 2	SEM, TEM	Spherical	30–40 nm	Vero cells	MTT assay, Trypan blue assay, Viral suppression experiments, cytopathic effect analysis	Inhibits virus replication	6.25- 100 μg/mL	[109]
Chemical synthesis (PVP coated)	HIV-1	SEM, TEM	Spherical	30–50 nm	HeLa-CD4-LTR-b-gal cells	Virus adsorption assays, HIV-1 infectivity inhibition assays, Cell-based fusion assay, HIV-1 gp120/CD4 ELISA Time-of-addition experiments, Virucidal activity assay	Interact with gp120 protein, inhibits binding and fusion	0.44 mg/mL (± 0.3)	[116]
Chemical synthesis (PVP-coated)	White Spot Syndrome Virus (WSSV)	TEM, FTIR, Zeta potential, UV–Vis	Spherical	1-90 nm	Shrimp (*P. vannamei*)	Histologic analysis, qRT-PCR WSSV-diagnosis, WSSV challenge bioassay,	Interfere with the viral envelopes	0.5–20 mg/mL	[132]
Chemical synthesis method (chitosan coated AgNPs)	H1N1 influenza virus	TEM, SEM, UV–Vis	Spherical	–	MDCK cells (Madin-darby canine kidney cells)	Antiviral assay	Damage virus protein	100 µg/mL	[123]
Chemical synthesis using Graphene oxide	Feline Coronavirus (Fcov)	HR-TEM, FE-SEM, XRD, XPS, AFM, TGA,	Spherical	5 and 25 nm	*Felis catus* whole fetus-4 (fcwf-4) cells	Tissue culture infectious dose (TCID) assay, Virus Inhibition Assay	Interferes with the lipid membrane of corona virus and ruptures it	0.1 mg/mL	[105]
Chemical synthesis using Graphene oxide	Infectious Bursal Disease Virus (IBDV)	HR-TEM, FE-SEM, XRD, XPS, AFM, TGA,	Spherical	5 and 25 nm	DF-1 cells	Tissue culture infectious dose (TCID) assay, Virus Inhibition Assay	Interferes with the viral protein	0.125 mg/mL	[105]
Chemical synthesis (Magnetic hybrid colloids- AgNPs)	Bacteriophage-X174, Murine norovirus (MNV), Adeno virus serotype2(Adv2)	SEM, TEM, EDS, ESEM	Spherical	7, 15, and 30 nm	-	Plaque assay, Real-time TaqMan PCR (RT-PCR) assays	Interact with viral protein, damage viral coating	57.5-400 ppm	[122, 133]
Electrochemical method	Poliovirus	FE-SEM, TEM, UV–Vis, EDX	Spherical	4 to 9 nm	Human rhabdomyosarcoma (ATCC # CCL-136) cells	MTT assay antiviral evaluation	Inhibits the polio virus particles	3.13 ppm	[134]
Essential oil reduction method using *Aquilaria sinensis* essential oil (AsEO)	Dengue and Zika viruses	SEM, TEM, EDS, XRD FTIR, U-Vis	Spherical	15 to 55 nm	*Aedes albopictus*	Histological studies	Severe destruction of midgut *Aedes albopictus*	44.23 to 166 ppm	[111]
Essential oil reduction method using *Pogostemon cablin* essential oil (PcEO)	Dengue and Zika viruses	SEM, TEM, EDS, XRD FTIR, U-Vis	Spherical	16 to 87 nm,	*Aedes albopictus*	Histological studies	Damage the digestive system of *Aedes albopictus*	32.49 to 90.05 ppm,	[111]
Green synthesis using *Andrographis paniculata* (AP-AgNPs)*, Phyllanthus niruri* (PN-AgNPs)*, Tinospora cordifolia* (TC-AgNPs)	Chikungunya virus	UV–Vis, SEM, XRD, FTIR, DLS, zeta potential,	Spherical	AP-AgNPs-70– 95 nm, PN-AgNPs-70 to 120 nm, TC-AgNPs-50–70 nm	Vero cells	Antiviral assay, Cytotoxicity assay, cytopathic effect, MNTD determination	Antiviral	MNTD-AP-AgNPs- 31.25 µg/ml PN-AgNPs- 125 µg/ml TC-AgNPs- 250 µg/ml	[112]
Green synthesis method using *Cinnamomum cassia*	H7N3 Influenza A Virus	UV–Vis, SEM, FTIR,	Spherical	25 to 55 nm	Vero cells	Cytotoxicity Assay- MTT, cytopathic effect analysis, Infectivity Assay,	Interferes with the protein, Cytotoxic	125 µg/ml	[135]
Green synthesis using Ginseng root extract	Influenza A virus	UV–Vis, XRD, FTIR, HR-TEM	Spherical	50 nm, 20 nm, and 2 nm	MDCK Cells	Antiviral assay- SRB assay (sulforhodamine B (SRB) assay)	Antiviral	0.02 and 0.25 M	[136]
Green synthesis using *Lampranthus coccineus & Malephora lutea* plant extract	HSV-1, HAV-10 virus & Coxsackie B4 virus	TEM, UV–Vis, FTIR	Spherical	10.12 nm to 27.89 nm	VERO cells	MTT assay, Metabolomics profiling (UPLC-MS) & molecular docking	Binds to viral envelop and inhibits penetration, interact with viral genome	5.13 µg/mL	[137]
Green synthesis using *Moringa oleifera* seed extract	Dengue virus (DEN-2)	UV–Vis, SEM, XRD, FTIR, EXD,	Spherical	100 nm	C6/36 and Vero cells	MTT, DEN-2 growth inhibition assays, Nanoparticle toxicity, plaque assay	Antiviral activity	10.24ppmto 21.17 ppm	[110]
Green synthesis using tannic acid	Herpes simplex virus 2 (HSV-2)	STEM, DLS, UV–Vis, EDX	Spherical	33 ± 7 nm	Mouse model	Flow Cytometry Phenotypic Analysis, Neutralization Assay, Quantitative Reverse Transcriptase-Polymerase Chain Reaction (qRT-PCR assays)	Act as bitter stimulant, increase immune cells,	5 µg/mL	[138]
Liquid-chemical synthesis technology	Kaposi’s sarcoma-associated herpesvirus (KSHV)	TEM	Spherical	5–200 nm	K-HeLa cells	Cytotoxicity assay, Apoptosis analysis, Reactive Oxygen Species Assay, Virion-cell binding and viral entry assay,	Increase ROS, destroy	< 0.6 μg/ml	[139]
Modified sonochemical reaction method	Herpes Simplex Virus Type 1	SEM, XPS, TGA	Spherical	4 ± 1 nm	Vero African green monkey kidney epithelial cells	Cell Toxicity Assay (XTT-based colorimetric assay), HSV-1 in vitro Assays, plaque reduction assay,	Cytopathic effect, interact with viral glycoprotein	200- 400 µg/mL	[119]
Monodisperse silica core silver nanoaprticles (chemical method)	Influenza A virus (IFV-A)	TEM, SEM, XPS,	Spherical	7–30 nm	MDCK	Plaque assay, real-time RT-PCR assay, ELISA, NA-Fluor Influenza Neuraminidase Assay, Flow cytometry analyses, hemagglutination assay	Interact with outer membrane of virus	-	[140]
Oxidation–reduction reaction	A/Human/Hubei/3/2005 Influenza Virus (H3N2 IFV)	TEM	Spherical	9.5 nm	MDCK cells	In vitro-MTT, hemagglutinin, flow cytometry, immunofluorescence	Damage the structure of virus, inhibit growth	ID_50_- 12.5 µg/mL	[118]
Chemical synthesis	H1V1 influenza virus	TEM	Spherical	5–20 nm	MDCK cells	Hemagglutination inhibition test, the embryo inoculation assay, Mosmann based MTT assay, flow cytometry assay	Ag + ion suppression respiration of pathogen, inhibitory action on virus	50 µg/mL	[141]
Turkevich method using aqueous trisodium citrate	HIV-1 pNL4.3-GFP + virus, HSV-1 and HSV-2	EDAX, SEM, FTIR, UV–Vis	Spherical	30–60 nm	293 T cells (Human embryonic kidney), (HeLa cell (human cervical cancer cells), HeLa-CD4-CCR5-LTR-β-gal cells, and Vero E6 cells (African green monkey kidney cells)	Anti-HIV infection assays, Anti-HSV infection assay, cell proliferation (WST-1) assay	Inactivate virus	–	[142]

## Therapeutic and future challenges of plant-based silver nanoparticles

Green synthesized AgNPs are the emerging area of research with enormous potent activity. Plant-derived phytoconstituents used for green synthesis, are the numerous sources of potent drug providing excellent activity to fight and destroy the devastating diseases like cancer and viral infection. The size, shape, and surface charge of AgNPs have a direct impact on their biological activity. Thus, complete profiling of pharmacodynamics and pharmacokinetics is needed to understand the exact mechanism, distribution, toxicity, and side-effects. Some limited controlled studies suggested the toxicity of AgNPs in macrophage immune cells, but there is a vast difference between in vitro and in vivo condition [143, 144].

After reviewing recent studies on AgNPs regarding cancer and viral infection leads to indicate some issues and limitations. (a) Detection of specific targets that AgNPs targets to kill the cancer cells to produce a targeted drug delivery system of AgNPs. (b) Identification of specific viruses against which AgNPs are efficiently potent. (c) Detection of specific combinations with which AgNPs show maximum potency for cancer and virus infection therapy. (d) Extensive studies are needed in vivo to develop clinically used AgNPs as a dosage form to treat the chronic disease like cancer. (e) The exact mechanism involved in the synthesis of green AgNPs is needed to be cleared. (f) Detailed studies on the toxicity of AgNPs in vivo is to be examined well.

Further many approaches can be utilized to increase the potency of nanoparticle of silver as by combining or hybridization. Magnetic hybrid colloid coated on AgNPs showed excellent results against specific viruses by inhibiting viral protein [122]. Viral infections whose mechanism is very typical to understand can be overpowered by using nano-sized particles. Several approaches to improve anticancer activity can also be made in nano-scale silver. Recently, a patent filed by Vijayan S. and Jisha MS reporting the antitumor and antimicrobial activity of bio-synthesized AgNPs using Withania endophyte *Colletotrichum gloeosporioides *conjugated with chitosan (patent publication number-2011841032445). Likewise, further work is necessary to achieve the optimum know how and understanding of AgNPs for different potent activity. AgNPs are prominent and can prove to be the boon in the field of nanotechnology by which excellent, effective, efficient and very potent nanoproduct can be formulated to treat the giant disease like cancer.

## Conclusion

This review comprises the therapeutic prospective of green synthesized AgNPs in the treatment of cancer and viral infections. Here, we first gave an overview of the green synthesis of AgNPs, then reviewed the applications of AgNPs in the treatment of cancer and their possible mechanism for cytotoxic activities. Further Phyto-based AgNPs with antiviral activity with their possible mechanism were discussed. Finally, some therapeutic and future challenges were summarized. Plant-based AgNPs have resulted in excellent biological activity with less toxicity to normal cells and highly toxic to cancerous cells. This makes the AgNPs as a promising candidate for future cancer treatment. AgNPs have also reported dominating activity against various life-threatening viruses that make them suitable for viral infection therapy.

Although various studies on size, shape, capping agenting, reducing agents of AgNPs have been performed, nevertheless there is still no clear optimum condition indicated for proper synthesis and development of target drug delivery system for cancer; thus, extensive studies are required in this field. In addition to this, long-term studies of AgNPs in vivo are necessary to evaluate the toxicity and performance.

## Data Availability

Data sharing is not applicable to this article.

## References

[1] Balantrapu K, Goia DV (2009). Silver nanoparticles for printable electronics and biological applications. J Mat Res.

[2] Hasan KMF, Pervez MN, Talukder ME, Sultana MZ, Mahmud S, Meraz MM, Bansal V, Genyang C (2019). A novel coloration of polyester fabric through green silver nanoparticles (G-AgNPs@PET). Nanomaterials.

[3] Burdusel AC, Gherasim O, Grumezescu AM, Mogoanta L, Ficai A, Andronescu E (2019). Biomedical applications of silver nanoparticles: an up-to-date overview. Mol.

[4] Karmous I, Pandey A, Ben K, Haj KB, Chaoui A (2020). Efficiency of the green synthesized nanoparticles as new tools in cancer therapy: insights on plant-based bioengineered nanoparticles, biophysical properties, and anticancer roles. Bio Tra Ele Res.

[5] Yesilot S, Aydin C (2019). Silver nanoparticles; a new hope in cancer therapy?. East J Med.

[6] Siadati SA, Afzali M, Sayadi M (2020). Could silver nano-particles control the 2019-nCoV virus? An urgent glance to the past. Chem Rev Lett.

[7] Rai M, Deshmukh SD, Ingle AP, Gupta IR, Galdiero M, Galdiero S (2016). Metal nanoparticles: the protective nano-shield against virus infection. Crit Rev Microbiol.

[8] Sondi I, Sondi BS (2004). Silver nanoparticles as antimicrobial agent: a case study on *E. coli* as a model for Gram-negative bacteria. J Col and Inter Sci.

[9] Nagarajan S, Kalaivani G, Poongothai E, Arul M, Natarajan H (2019). Characterization of silver nanoparticles synthesized from *Catharanthus roseus* (*Vinca rosea*) plant leaf extract and their antibacterial activity. IJRAR.

[10] Al-Shmgani HSA, Mohammed WH, Sulaiman GM, Saadoon AH (2017). Biosynthesis of Silver nanoparticles from *Catharanthus roseus* leaf extract and assessing their antioxidant, antimicrobial, and wound-healing activities. Art Cell Nanomed Biotech.

[11] Deya C, Bellotti N (2017). Biosynthesized silver nanoparticles to control fungal infections in indoor environments. Adv Nat Sci Nanosci Nanotechnol.

[12] Singh P, Ahn S, Kang JP, Veronika S, Huo Y, Singh H, Chokkaligam M, El-Agamy Farh M, Aceituno VC, Kim YJ, Yang DC (2018). In vitro anti-inflammatory activity of spherical silver nanoparticles and monodisperse hexagonal gold nanoparticles by fruit extract of *Prunus serrulata*: a green synthetic approach. Artific Cells Nanomed Biotechnol.

[13] Yuan YG, Zhang S, Hwang JY, Kong IK (2018). Silver nanoparticles potentiates cytotoxicity and apoptotic potential of camptothecin in human cervical cancer cells. Oxida Medi Cellu Longe.

[14] Kalishwaralal K, Banumathi E, Pandian SRK, Deepak V, Muniyandi J (2009). Silver nanoparticles inhibit VEGF induced cell proliferation and migration in bovine retinal endothelial cells. Coll and Surf B: Biointer.

[15] Nadaroglu H, Alayli GA, Ince S (2017). Synthesis of nanoparticles by green synthesis method. Int J Inno Res Rev.

[16] Herlekar M, Barve S, Kumar R (2014). Plant-mediated green synthesis of iron nanoparticles. J Nanoparti..

[17] Mousavi B, Tafvizi F, Bostanabad SZ (2018). Green synthesis of silver nanoparticles using *Artemisia turcomanica* leaf extract and the study of anti-cancer effect and apoptosis induction on gastric cancer cell line (AGS). Artifi Cells Nanomed Biotech.

[18] Thakur S, Mohan GK (2019). Green synthesis of silver nanoparticles of boswellic acid, and it’s in vitro anticancer activity. Int J Pharma Bio Sci.

[19] Bedlovicova Z, Salayova A (2017). Green-Synthesized Silver Nanoparticles and Their Potential for Antibacterial Applications. Bacterial Pathogenesis Antibacterial Control..

[20] Silva LP, Pereira TM, Bonatto CC (2019). Frontiers and perspectives in the green synthesis of silver nanoparticles. Green Synth Characterizat Applicat Nanoparticles..

[21] Sanjay SS (2019). Safe nano is green nano. Green Synth Characterizat Applicat Nanoparticles.

[22] Ghosh S (2019). Green synthesis of nanoparticles and fungal infection. Green Synth Characterizat Applicat Nanoparticles.

[23] Roy A, Bulut O, Some S, Mandal AK, Yilmaz MD (2019). Green synthesis of silver nanoparticles: biomolecule-nanoparticle organizations targeting antimicrobial activity. RSC Adv.

[24] Devatha CP, Thalla AK (2018). Green synthesis of nanomaterials. Synthe Inorganic Nanomater.

[25] Jang SJ, Yang IJ, Tettey CO, Kim KM, Shin HM (2016). In-vitro anticancer activity of green synthesized silver nanoparticles on MCF-7 human breast cancer cells. Mat Sci Engineer C.

[26] Majeed S, Bakhtiar NFB, Danish M, Mohamad Ibrahim MN, Hashim R (2019). Green approach for the biosynthesis of silver nanoparticles and its antibacterial and antitumor effect against osteoblast MG-63 and breast MCF-7 cancer cell lines. Sus Chem Pharma.

[27] Krithiga N, Rajalakshmi N, Jayachitra A (2015). Green synthesis of silver nanoparticles using leaf extracts of *Clitoria ternatea* and *Solanum nigrum* and study of its antibacterial effect against common nosocomial pathogens. J Nanosci.

[28] Mollick MMR, Rana D, Dash SK, Chattopadhyay S, Bhowmick B, Maitya D, Mondala D (2019). Studies on green synthesized silver nanoparticles using *Abelmoschus esculentus* (L.) pulp extract having anticancer (in vitro) and antimicrobial applications. Ara J Chem.

[29] Chen X, Jensen L (2016). Understanding the shape effect on the plasmonic response of small ligand coated nanoparticles. J Opt..

[30] Noah N (2019). Green synthesis: characterization and application of silver and gold nanoparticles. Green Synth Characterizat Applicat Nanoparticles..

[31] Khan MJ, Shameli K, Sazili AQ, Selamat J, Kumari S (2019). Rapid green synthesis and characterization of silver nanoparticles arbitrated by curcumin in an alkaline medium. Molecules.

[32] Pirtarighat S, Ghannadnia M, Baghshahi S (2019). Green synthesis of silver nanoparticles using the plant extract of *Salvia spinosa* grown in vitro and their antibacterial activity assessment. J Nanostruc Chemis.

[33] Gudikandula K, Maringanti SC (2016). Synthesis of silver nanoparticles by chemical and biological methods and their antimicrobial properties. J Exp Nanosci.

[34] Ahmed S, Saifullah Ahmad M, Swami BL, Ikram S (2016). Green synthesis of silver nanoparticles using *Azadirachta indica* aqueous leaf extract. J Rad Res App Sci.

[35] Osibe DA, Chiejina NV, Ogawa K, Aoyagi H (2018). Stable antibacterial silver nanoparticles produced with seed-derived callus extract of *Catharanthus roseus*. Art Cells Nanomed Biotech.

[36] Mukunthan KS, Elumalai EK, Patel TN, Murty RV (2011). *Catharanthus roseus*: a natural source for the synthesis of silver nanoparticles. Asian Pac J Trop Biomed.

[37] Jyoti K, Baunthiya M, Singh A (2016). Characterization of silver nanoparticles synthesized using *Urtica dioica* Linn. leaves and their synergistic effects with antibiotics. J Rad Res App Sci.

[38] Anandalakshmi K, Venugob J, Ramasamy V (2016). Characterization of silver nanoparticles by green synthesis method using *Pedalium murex* leaf extract and their antibacterial activity. App Nanosci.

[39] Baudot C, Tan CM, Kong JC (2010). FTIR spectroscopy as a tool for nano-material characterization. Infrared Phys Technol.

[40] Rautela A, Rani J, Das MD (2019). Green synthesis of silver nanoparticles from *Tectona grandis* seeds extract: characterization and mechanism of antimicrobial action on different microorganisms. J Ana Sci Tech.

[41] Khan A, Al-Harrasi A, Rehman NU, Sarwar R, Ahmad T, Ghaffar R, Khan H, Al-Amri I, Csuk R, Al-Rawahi A (2019). Loading AKBA on surface of silver nanoparticles to improve their sedative-hypnotic and anti-inflammatory efficacies. Nanomed..

[42] Sivalingam D, Karthikeyan S, Arumugam P (2012). Biosynthesis of silver nanoparticles from *Glycyrrhiza glabra* root extract. Arch App Sci Res.

[43] Shafaghat A (2015). Synthesis and characterization of silver nanoparticles by photosynthesis method and their biological activity, synthesis and reactivity in inorganic. Metal-Org Nano-Met Chem.

[44] Devaraj P, Kumari P, Aarti C, Renganathan A (2013). Synthesis and characterization of silver nanoparticles using cannonball leaves and their cytotoxic activity against MCF-7 Cell Line. J Nanotech..

[45] Srirangam GM, Rao KP (2017). Synthesis and characterization of silver nanoparticles from the leaf extract of Malachra capitata (l). Ras J Chem.

[46] Garibo D, Borbon-Nunez HA, de Leon JND, Mendoza EG, Estrada I, Toledano-Magana Y, Tiznado H, Ovalle-Marroquin M, Soto-Ramos AG, Blanco A, Rodríguez JA (2020). Green synthesis of silver nanoparticles using *Lysiloma acapulcensis* exhibit high-antimicrobial activity. Scienti rep.

[47] Rasheed M, Ali A, Kanwal S, Ismail M, Sabir N, Amin F (2019). Synergy of green tea reduced tamoxifen-loaded silver nanoparticles exhibit OGT downregulation in breast cancer cell line. Dig J Nanomat Biostr.

[48] Gurunathan S, Qasim M, Park C, Yoo H, Kim IDJH, Hong K (2018). Cytotoxic potential and molecular pathway analysis of silver nanoparticles in human colon cancer cells HCT116. Int J Mol Sci.

[49] Zhang K, Liu X, Samson OASR, Ramachandran AK, Ibrahim IAA, Nassir AM, Yao J (2019). Synthesis of silver nanoparticles (AgNPs) from leaf extract of *Salvia miltiorrhiza* and its anticancer potential in human prostate cancer LNCaP cell lines. Art Cells Nanomed Biotech.

[50] Usmani A, Mishra A, Jafri A, Arshad M, Siddiqui MA (2019). Green synthesis of silver nanocomposites of *Nigella sativa* seeds extract for hepatocellular carcinoma. Cur Nanomat.

[51] Muthukrishnan S, Vellingiri B, Murugesan G (2018). Anticancer effects of silver nanoparticles encapsulated by *Gloriosa superba* (L.) leaf extracts in DLA tumor cells. Fut J Pharma Sci.

[52] Erdogan O, Abbak M, Demirbolat GM, Birtekocak F, Aksel M, Pasa S (2019). Green synthesis of silver nanoparticles via *Cynara scolymus* leaf extracts: The characterization, anticancer potential with photodynamic therapy in MCF7 cells. PLoS ONE.

[53] Carson L, Bandara S, Joseph M, Green T, Grady T, Osuji G, Weerasooriya A, Ampim P, Woldesenbet S (2020). Green synthesis of silver nanoparticles with antimicrobial properties using *Phyla dulcis* plant extract. Foodbor patho dis.

[54] Kumari R, Saini AK, Kumar A, Saini RV (2020). Apoptosis induction in lung and prostate cancer cells through silver nanoparticles synthesized from *Pinus roxburghii* bioactive fraction. J Bio Inorg Chem.

[55] Singh D, Yadav E, Falls N, Kumar V, Singh M, Verma A (2019). Phyto-fabricated silver nanoparticles of *Phyllanthus emblica* attenuated diethyl-nitrosamine-induced hepatic cancer via knock-down oxidative stress and inflammation. Inflammopharmacol.

[56] Yusuf A, Casey A (2020). Liposomal encapsulation of silver nanoparticles (AgNP) improved nanoparticle uptake and induced redox imbalance to activate caspase-dependent apoptosis. Apo.

[57] El-Naggar NE, Hussein MH, El-Sawah AA (2017). Bio-fabrication of silver nanoparticles by phycocyanin, characterization, in vitro anticancer activity against breast cancer cell line and in vivo cytotoxicity. Scient Rep.

[58] Simard JC, Durocher I, Girard D (2016). Silver nanoparticles induce irremediable endoplasmic reticulum stress leading to unfolded protein response dependent apoptosis in breast cancer cells. Apo.

[59] Lee YS, Kim DW, Lee YH, Oh JH, Yoon S, Choi MS, Lee SK, Kim JW, Lee K, Song CW (2011). Silver nanoparticles induce apoptosis and G2/M arrest via PKC_f_-dependent signaling in A549 lung cells. Arch Toxicol.

[60] Vasanth K, Ilango K, Kumar MR, Agrawal A, Dubey GP (2014). Anticancer activity of *Moringa oleifera* mediated silver nanoparticles on human cervical carcinoma cells by apoptosis. Coll Surf B: Bioint.

[61] Valsalam S, Paul A, Arasu MV, Al-Dhabi NA, Mohammed Ghilan AK, Kaviyarasu K, Ravindran B, Chang SW, Arokiyaraj S (2018). Rapid biosynthesis and characterization of silver nanoparticles from the leaf extract of *Tropaeolum majus* L. and its enhanced in-vitro antibacterial, antifungal, antioxidant and anticancer properties. J Photochem Photobiol, B.

[62] Sarkar S, Kotteeswara V (2018). Green synthesis of silver nanoparticles from aqueous leaf extract of Pomegranate (*Punica granatum*) and their anticancer activity on human cervical cancer cells. Adv Nat Sci Nanosci Nanotechnol.

[63] Hashemi F, Tasharrofi N, Saber MM (2020). Green synthesis of silver nanoparticles using *Teucrium polium* leaf extract and assessment of their antitumor effects against MNK45 human gastric cancer cell line. J Mol Str.

[64] Wang L, Xu J, Yan Y, Liu H, Karunakaran T, Li F (2019). Green synthesis of gold nanoparticles from *Scutellaria barbata* and its anticancer activity in pancreatic cancer cell (PANC-1). Art Cells Nanomed Biotech.

[65] Foldbjerg R, Dang AD, Autrup H (2011). Cytotoxicity and genotoxicity of silver nanoparticles in the human lung cancer cell line, A549. Arch Toxicol.

[66] Blanco J, Lafuente D, Gómez T, García T, Domingo JL, Sánchez DJ (2017). Polyvinyl pyrrolidone-coated silver nanoparticles in a human lung cancer cells: time-and dose-dependent influence over p53 and caspase-3 protein expression and epigenetic effects. Arch Toxicol.

[67] Kuppusamy P, Ichwan SJA, Al-Zikri PNH, Suriyah WH, Soundharrajan I, Govindan N, Yusoff MM, (2016) In Vitro Anticancer Activity of Au, Ag Nanoparticles Synthesized Using *Commelina nudiflora* L. Aqueous Extract Against HCT-116 Colon Cancer Cells. Bio Tr El Res 173(2):297–305 10.1007/s12011-016-0666-7 26961292

[68] Karthik S, Sankar R, Varunkumar K, Ravikumar V (2014). Romidepsin induces cell cycle arrest, apoptosis, histone hyperacetylation and reduces matrix metalloproteinases 2 and 9 expression in bortezomib sensitized non-small cell lung cancer cells. Biomed Pharmacother.

[69] Nair APV, Sethu S, Lim HK, Balaji G, Valiyaveettil G, Hande MP (2012). Differential regulation of intracellular factors mediating cell cycle, DNA repair and inflammation following exposure to silver nanoparticles in human cells. Gen Int.

[70] Pei J, Fu B, Jiang L, Sun T (2019). Biosynthesis, characterization, and anticancer effect of plant-mediated silver nanoparticles using *Coptis chinensis*. Int J Nanomed.

[71] Jeng PS, Inoue-Yamauchi A, Hsieh JJ, Cheng EH (2018). BH3-dependent and independent activation of BAX and BAK in mitochondrial apoptosis. Cur Op Physio.

[72] Siddiqui WA, Ahad A, Ahsan H (2015). The mystery of BCL2 family: Bcl-2 proteins and apoptosis: an update. Archi Toxicol.

[73] Yang T, Yao Q, Cao F, Liu Q, Liu B, Wang XH (2016). Silver nanoparticles inhibit the function of hypoxia-inducible factor-1 and target genes: insight into the cytotoxicity and anti-angiogenesis. Int J Nanomed.

[74] Gurunathan S, Lee KJ, Kalishwaralal K, Sheikpranbabu S, Vaidyanathan R, Eom SH (2009). Antiangiogenic properties of silver nanoparticles. Biomaterial.

[75] Vivek R, Thangam R, Muthuchelian K, Gunasekaran P, Kaveri K, Kannan S (2012). Green biosynthesis of silver nanoparticles from *Annona squamosa* leaf extract and it’s in vitro cytotoxic effect on MCF-7 cells. Pro Biochem.

[76] Huo Y, Singh P, Kim YJ, Soshnikov V, Kang J, Markus J (2018). Biological synthesis of gold and silver chloride nanoparticles by *Glycyrrhiza uralensis* and invitro applications. Art Cell Nanomed Biotech.

[77] Khorrami S, Zarrabi A, Khaleghi M, Danaei M, Mozafari M (2018). Selective cytotoxicity of green synthesized silver nanoparticles against the McF-7 tumor cell line and their enhanced antioxidant and antimicrobial properties. Int J Nanomed.

[78] Jeyaraja A, Sathishkumara G, Sivanandhana G, MubarakAlid D, Rajesha M, Arun R (2013). Biogenic silver nanoparticles for cancer treatment: an experimental report. Col Sur Biointer.

[79] Kathiravan V, Ravi S, Ashok kumar S, (2014). Synthesis of silver nanoparticles from *Melia dubia* leaf extract and their in vitro anticancer activity. Spectro Acta Part A: Mol Biomol Spectro.

[80] Firdhouse MJ, Lalith P (2013). Biosynthesis of silver nanoparticles using the extract of *Alternanthera sessilis*—antiproliferative effect against prostate cancer cells. Cancer Nano.

[81] Mukundan D, Mohan kumar R, Vasanth kumari R (2015). Green synthesis of silver nanoparticles using leaves extract of *Bauhinia Tomentosa* linn and its invitro anticancer potential. Mater Today Proceed.

[82] Nilavukkarasia M, Vijayakumar S, Kumar SP (2020). Biological synthesis and characterization of silver nanoparticles with *Capparis zeylanica* L. leaf extract for potent antimicrobial and anti-proliferation efficiency. Mate Sci for Ene Techno.

[83] Vijayan R, Joseph S, Mathew B (2018). *Indigofera tinctoria* leaf extract mediated green synthesis of silver and gold nanoparticles and assessment of their anticancer, antimicrobial, antioxidant and catalytic properties. Art Cells Nanomed Biotech.

[84] Nayaka S, Chakraborty B, Pallavi SS, Bhat MP, Shashiraj KN, Ghasti B (2020). Synthesis of biogenic silver nanoparticles using *Zanthoxylum rhetsa* (Roxb) DC seed coat extract as reducing agent and in - vitro assessment of anticancer effect on A549 lung cancer cell line. Int J Pharma Res.

[85] Satsangi N (2020). Synthesis and characterization of biocompatible silver nanoparticles for anticancer application. J Inorg and Organometal Pol and Mat.

[86] Ali Abuderman A, Syed RA, Alyousef AS, Alqahtani M, Shamsul Ola M, Malik A (2019). Green synthesized silver Nanoparticles of *Myrtus communis* L (AgMC) extract inhibits cancer hallmarks via targeting aldose reductase (AR) and associated signaling network. PRO.

[87] Saratale RG, Benelli G, Kumar G, Kim DS, Saratale GD (2017). Bio-fabrication of silver nanoparticles using the leaf extract of an ancient herbal medicine, dandelion (*Taraxacum officinale*), evaluation of their antioxidant, anticancer potential, and antimicrobial activity against phytopathogens. Environ Sci Poll Res.

[88] Das S, Das J, Samadder A, Bhattacharyya SS, Das D, Khuda-Bukhsh AR (2013). Biosynthesized silver nanoparticles by ethanolic extracts of *Phytolacca decandra, Gelsemium sempervirens, Hydrastis canadensis* and *Thuja occidentalis* induce differential cytotoxicity through G2/M arrest in A375 cells. Col Sur B: Bioin.

[89] Dipankar C, Murugan S (2012). The green synthesis, characterization and evaluation of the biological activities of silver nanoparticles synthesized from *Iresine herbstii* leaf aqueous extracts. Col Sur: Biointer.

[90] Al-Sheddi ES, Farshori NN, Al-Oqail MM, Al-Massarani SM, Saquib Q, Wahab R (2018). Anticancer potential of green synthesized silver nanoparticles using extract of *Nepeta deflersiana* against human cervical cancer cells (HeLA). Bioinorg Chem App..

[91] Mahendran G, Kumari BDR (2016). Biological Activities of silver nanoparticles from *Nothapodytes nimmonian* (Graham) Mabb. Fruit extracts. F Sci and Hu Well.

[92] Islam NU, Amin R, Shahid M, Amin M, Zaib S, Iqbal I (2017). A multi-target therapeutic potential of *Prunus domestica* gum stabilized nanoparticles exhibited prospective anticancer, antibacterial, urease-inhibition, anti-inflammatory and analgesic properties. BMC Comp Alt Med.

[93] Prabhu D, Arulvasu C, Babu G, Manikandan R, Srinivasan P (2013). Biologically synthesized green silver nanoparticles from leaf extract of *Vitex negundo L.* induce growth-inhibitory effect on human colon cancer cell line HCT15. Pro Biochem.

[94] Hemlata Meena PR, Singh AP, Tejavath KK (2020). Biosynthesis of silver nanoparticles using *Cucumis prophetarum* aqueous leaf extract and their antibacterial and antiproliferative activity against cancer cell lines. ACS Omega.

[95] Sriranjani R, Srinithyaa S, Vellingiri V, Brindha P, Anthony SP, Sivasubramanian A, Muthuramana MS (2016). Silver nanoparticle synthesis using *Clerodendrum phlomidis* leaf extract and preliminary investigation of its antioxidant and anticancer activities. J Mol Li.

[96] Suwannakul S, Wacharanad S, Chaibenjawong P (2018). Rapid green synthesis of silver nanoparticles and evaluation of their properties for oral disease therapy. Sci Techno.

[97] Pandian S, Chidambaram S (2017). Antimicrobial, cytotoxicity and anti-cancer activity of silver nanoparticles from *Glycyrrhiza glabra*. IJPSR.

[98] Botcha S, Prattipati SD (2020). Callus extract mediated green synthesis of silver nanoparticles, their characterization and cytotoxicity evaluation against MDA-MB-231 and PC-3 Cells. Bio Nano Sci.

[99] Ahmed MJ, Murtaza G, Rashid F, Iqbal J (2019). Eco-friendly green synthesis of silver nanoparticles and their potential applications as antioxidant and anticancer agents. Drug Dev Ind Pharm.

[100] Sukirtha R, Priyanka KM, Antony JJ, Kamalakkannan S, Thangam R, Gunasekaran P, Achiraman S (2012). Cytotoxic effect of Green synthesized silver nanoparticles using *Melia azedarach* against in vitro HeLa cell lines and lymphoma mice model. Pro Biochem.

[101] Hemalatha KPJ, Shantakani S, Botcha S (2019). Green synthesis of silver nanoparticles using aqueous fruit and tuber extracts of *Momordica cymbalaria*. J Plant Biochem Biotech..

[102] Chahardoli A, Karimi N, Fattahi A (2017). Biosynthesis, characterization, antimicrobial and cytotoxic effects of silver nanoparticles using *Nigella arvensis* seed extract. Ira J Pharma Res.

[103] Satpathy S, Patra A, Ahirwar B, Hussain MD (2018). Antioxidant and anticancer activities of green synthesized silver nanoparticles using aqueous extract of tubers of *Pueraria tuberosa*. Art Cell Nanomed Biotech.

[104] Young Ahn E, Jin H, Park Y (2019). Green synthesis and biological activities of silver nanoparticles prepared by *Carpesium cernuum* extract. Arch Pharm Res.

[105] Chen YN, Hsueh YH, Hsieh CT, Tzou DY, Chang PL (2016). Antiviral activity of graphene-silver nanocomposites against non-enveloped and enveloped viruses. Int J Environ Res.

[106] Kim M, Nguyen DY, Heo Y, Park KH, Paik HD, Kim YB (2020). Antiviral activity of *Fritillaria thunbergii* extract against Human Influenza Virus H1N1 (PR8) In Vitro, In Ovo and In Vivo. J Microbiol Biotechnol.

[107] Elechiguerra JL, Burt JL, Morones JR, Bragado BC, Gao X, Lara HH, Yacaman MJ (2005). Interaction of silver nanoparticles with HIV-1. J Nanobiotech.

[108] Chen N, Zheng Y, Yina J, Lia X, Zhenga C (2013). Inhibitory effects of silver nanoparticles against adenovirus type 3 in vitro. J Vir Met.

[109] Hu RL, Li SR, Kong FJ, Hou RJ, Guan XL, Guo F (2014). Inhibition effect of silver nanoparticles on herpes simplex virus 2. Gen Mol Res.

[110] Sujitha V, Murugan K, Paulpandi K, Panneerselvam C, Suresh U, Roni M, Nicoletti M (2015). Green-synthesized silver nanoparticles as a novel control tool against dengue virus (DEN-2) and its primary vector *Aedes aegypti*. Parasitol Res.

[111] Gaal H, Fouad H, Mao G, Tian J, Jianchu M (2017). Larvicidal and pupicidal evaluation of silver nanoparticles synthesized using *Aquilaria sinensis* and *Pogostemon cablin* essential oils against dengue and zika viruses’ vector *Aedes albopictus* mosquito and its histopathological analysis. Art Cell Nanomed Biotech.

[112] Sharma V, Kaushik S, Pandit P, Dhull D, Yadav JP, Kaushik S (2019). Green synthesis of silver nanoparticles from medicinal plants and evaluation of their antiviral potential against chikungunya virus. App Microbio Biotech.

[113] Bekele AZ, Gokulan K, Williams KM, Khare S (2016). Dose and size-AU2 c dependent antiviral effects of silver nanoparticles on feline calicivirus, a human norovirus surrogate. Foodbor Patho Dis.

[114] El-Mohamady RS, Ghattas TA, Zawrah MF, Abd El-Hafeiz YGM (2018). Inhibitory effect of silver nanoparticles on bovine herpesvirus-1. Int J Vet Sci Med.

[115] Gaikwad S, Ingle A, Gade A, Rai M, Falanga A, Incoronato N, Russo L, Galdiero S, Galdiero M (2013). Antiviral activity of myco-synthesized silver nanoparticles against herpes simplex virus and human parainfluenza virus type 3. Int J Nanomed.

[116] Lara HH, Ayala-Nuñez AV, Ixtepan-Turrent L, Rodriguez-Padill C (2010). Mode of antiviral action of silver nanoparticles against HIV-1. J Nanobiotech.

[117] Speshock JL, Murdock RC, Braydich-Stolle LK, Schrand AM, Hussain SM (2010). Interaction of silver nanoparticles with Tacaribe virus. J Nanobiotech.

[118] Xiang D, Zheng Y, Duan W, Li X, Yin J, Shigdar S, Liam M (2013). Inhibition of a/human/hubei/3/2005 (h3N2) influenza virus infection by silver nanoparticles in vitro and in vivo. Int J Nanomed.

[119] Baram-Pinto D, Shukla S, Perkas N, Gedanken A, Sarid R (2009). Inhibition of herpes simplex virus type 1 infection by silver nanoparticles capped with mercaptoethane sulfonate. Bioconjugate Chem.

[120] Avilala J, Golla N (2019). Antibacterial and antiviral properties of silver nanoparticles synthesized by marine actinomycetes. IJPSR.

[121] Yang XX, Li CM, Huang CZ (2016). Curcumin modified silver nanoparticles for highly efficient inhibition of respiratory syncytial virus infection. Nanoscale.

[122] Park SJ, Park HH, Kim SY, Kim SJ, Woo K, Ko GP (2014). Antiviral properties of silver nanoparticles on a magnetic hybrid colloid. App Environ Microbio.

[123] Mori Y, Ono T, Miyahira Y, Nguyen VQ, Matsui T, Ishihara M (2013). Antiviral activity of silver nanoparticle/chitosan composites against H1N1 influenza a virus. Nanosca Res Let.

[124] Dhanasezhian A, Srivani S, Govindaraju K, Parija P, Sasikala S, Kumar MRR (2019). Anti-Herpes Simplex Virus (HSV-1 and HSV-2) activity of biogenic gold and silver nanoparticles using seaweed *Sargassum wightii*. Ind J Geo Mar Sci.

[125] Govindaraju K, Kiruthiga V, Kumar VG, Singaravelu G (2009). Extracellular synthesis of silver nanoparticles by a marine alga, *Sargassum wightii* grevilli and their antibacterial effects. J Nanosci Nanotech.

[126] Elbeshehy EKF, Elazzazy AM, Aggelis G (2015). Silver nanoparticles synthesis mediated by new isolates of *Bacillus* spp., nanoparticle characterization and their activity against Bean Yellow Mosaic Virus and human pathogens. Front Microbio.

[127] Tamilselvan S, Ak T, Kasivelu G (2017). Microscopy based studies on the interaction of bio-based silver nanoparticles with Bombyx mori Nuclear Polyhedrosis virus. J Virologic Met.

[128] Trefry JC, Wooley DP (2013). Silver nanoparticles inhibit vaccinia virus infection by preventing viral entry through a macropinocytosis-dependent mechanism. J Biomed Nanotech.

[129] Omara ST, Zawrah MF, Samy AA (2017). Minimum bactericidal concentration of chemically synthesized silver nanoparticles against pathogenic Salmonella and Shigella strains isolated from layer poultry farms. J App Pharma Sci.

[130] Dung TTN, Nam VN, Nhan TT, Ngoc TTB, Minh LQ, Nga BTT, Le VP, Quang DV (2020). Silver nanoparticles as potential antiviral agents against African swine fever virus. Mat Res Ex.

[131] Morris D, Ansar M, Speshock J, Ivanciuc T, Qu Y, Casola A, Garofalo R (2019). Antiviral and immunomodulatory activity of silver nanoparticles in experimental RSV infection. Vir.

[132] Ochoa-Meza AR, Álvarez-Sánchez AR, Romo-Quiñonez CR, Barraza A, Magallón-Barajas FJ, Chávez-Sánchez A (2019). Silver nanoparticles enhance survival of white spot syndrome virus infected *Penaeus vannamei* shrimps by activation of its immunological system. Fish Shellfish Immuno.

[133] Park HH, Park S, Ko G, Woo K (2013). Magnetic hybrid colloids decorated with Ag nanoparticles bite away bacteria and chemisorb viruses. J Mat Chem B.

[134] Quang HT, Thanh H, Thi N, Thuy Thanh N, Van Chung P, Hung Ngoc P (2017). Cytotoxicity and antiviral activity of electrochemical−synthesized silver nanoparticles against poliovirus. J Virologic Met.

[135] Fatima M, Zaidi NSS, Amraiz D, Afzal F (2016). In vitro antiviral activity of *Cinnamomum cassia* and its nanoparticles against H7N3 influenza a virus. J Microbiol Biotechnol.

[136] Sreekanth TVM, Nagajyothi PC, Muthuraman P, Enkhtaivan G, Vattikuti SVP, Tettey CO (2018). Ultra-sonication-assisted silver nanoparticles using *Panax ginseng* root extract and their anti-cancer and antiviral activities. J Photochem Photobio B Bio..

[137] Haggag EG, Elshamy AM, Rabeh MA, Gabr NM, Salem M, Youssif KA, Samir S (2019). Antiviral potential of green synthesized silver nanoparticles of *Lampranthus coccineus* and *Malephora lute*. Int J Nanomed.

[138] Orlowski P, Kowalczyk A, Tomaszewska E, Ranoszek-Soliwoda K, We˛grzyn A, Grzesiak J, Celichowski G (2018). Antiviral activity of tannic acid modified silver nanoparticles: potential to activate immune response in herpes genitalis. Virus.

[139] Wan C, Tai J, Zhang J, Guo Y, Zhu Q, Ling D (2019). Silver nanoparticles selectively induce human oncogenic γ-herpesvirus-related cancer cell death through reactivating viral lytic replication. Cell Death Dis.

[140] Park SJ, Ko YS, Lee SJ, Lee C, Woo K, Ko GP (2018). Inactivation of influenza A virus via exposure to silver nanoparticle-decorated silica hybrid composites. Environ Sci Poll Res..

[141] Xiang D, Chen Q, Pang L, Zheng C (2011). Inhibitory effects of Silver nanoparticles on H1N1 influenza A virus in vitro. J Virologic Met.

[142] Fayaz AM, Ao Z, Girilal M, Chen L, Xiao X, Kalaichelvan PT, Yao X (2012). Inactivation of microbial infectiousness by silver nanoparticles-coated condom: a new approach to inhibit HIV- and HSV-transmitted infection. Int J Nanomed.

[143] Ge L, Li Q, Wang M, Ouyang J, Li X, Xing MMQ (2014). Nanosilver particles in medical applications: synthesis, performance, and toxicity. Int J Nanomed.

[144] Park J, Lim DH, Lim HJ, Kwon T, Choi J, Jeong S, Choi I, Cheon J (2011). Size dependent macrophage responses and toxicological effects of Ag nanoparticles. Chem Commun.

